# On the cause of low thermal stability of ethyl halodiazoacetates

**DOI:** 10.3762/bjoc.12.155

**Published:** 2016-07-26

**Authors:** Magnus Mortén, Martin Hennum, Tore Bonge-Hansen

**Affiliations:** 1 Department of Chemistry, University of Oslo, P.O. Box 1033 Blindern, NO-0315 Oslo, Norway

**Keywords:** carbenes, catalysis, cyclopropanation, halo diazoacetates, half-lives, thermal stability

## Abstract

Rates for the thermal decomposition of ethyl halodiazoacetates (halo = Cl, Br, I) have been obtained, and reported herein are their half-lives. The experimental results are supported by DFT calculations, and we provide a possible explanation for the reduced thermal stability of ethyl halodiazoacetates compared to ethyl diazoacetate and for the relative decomposition rates between the chloro, bromo and iodo analogs. We have also briefly studied the thermal, non-catalytic cyclopropanation of styrenes and compared the results to the analogous Rh(II)-catalyzed reactions.

## Introduction

**Figure 1:**
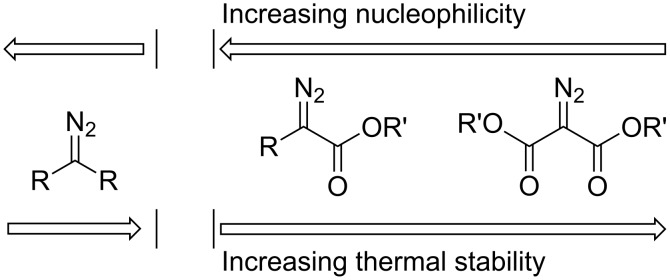
Relative stability and nucleophilicity of non-stabilized (R = H, alkyl) diazo compounds (left) and stabilized (R’ = alkyl, R = H, alkyl) diazo esters (right).

The chemistry of diazo compounds has fascinated organic chemists ever since Theodor Curtius synthesized ethyl diazoacetate (EDA, **1**) for the first time in 1883 [1]. Even today, after more than a century of research, diazo compounds still play an important role in state-of-the-art organic chemistry in areas such as for example C–H functionalization [2]. The synthesis and properties of diazo compounds have been a topic of much interest, particularly relevant are their thermal stability and sensitivity towards Brønsted and Lewis acids. The monograph by Regitz and Maas gives an excellent overview on their preparation and properties [3]. The thermal stability of diazo compounds is highly influenced by the α-substituents present in their molecular structure. A simplified illustration of the thermal stabilities for some selected classes of diazo compounds is shown in Figure 1.

Aliphatic, non-stabilized diazo compounds are thermally labile and usually decompose within hours at room temperature. They are inherently unstable to acid and the diazo carbon has a significant nucleophilic character. Even though a complete understanding is lacking for the thermal decomposition of aliphatic diazo compounds, the increased stability of diazo carbonyl compounds relative to their aliphatic counterparts is explained by the electron-acceptor character of the carbonyl group. The presence of one or two ester groups α to the diazo functionality leads to increased stability so that elevated temperatures are usually needed in order to induce thermal decomposition. Ethyl diazoacetate (EDA) is relatively safe to handle, being thermally stable at room temperature with a reported half-life of 109 hours at 100 °C [3]. EDA is also stable in weakly acidic solutions such as glacial acid [4], but reactive in the presence of Lewis acids, a key property for its effectiveness in transition metal-catalyzed reactions [5]. A large number of diazo esters have been synthesized and an order has been arranged for α-substituents that increase the stability of the diazoesters [3]. Halogen substituents are completely absent from this list of α-substituents. Hence, the relative position of halodiazoesters in Figure 1 is yet unknown.

**Scheme 1:**
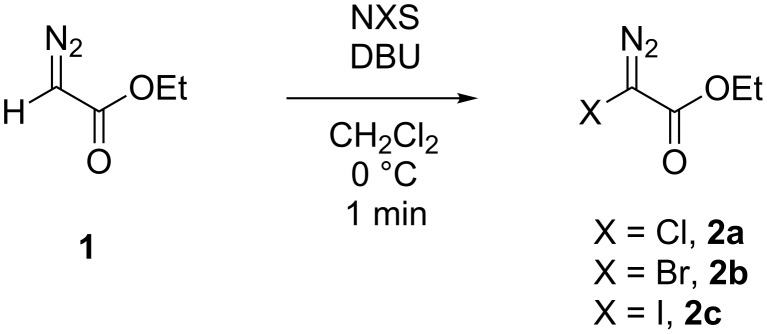
Synthesis of ethyl halodiazoacetates [11].

The first syntheses of halodiazoacetates were described in the literature in the late 1960s by Schöllkopf and co-workers [6–10], but no stability data was reported. We later developed a rapid and efficient synthetic procedure for the synthesis of ethyl halodiazoacetates **2a**–**c** (Scheme 1) from **1** and studied their reactivity in Rh(II)-catalyzed reactions [11]. In the presence of Rh(II) catalysts the halodiazoacetates extrude N_2_ and form the corresponding Rh–carbenes which undergo typical carbenoid reactions such as cyclopropanation [11], cyclopropanation–ring expansion [12], and C–H insertion and Si–H insertion reactions [13].

Under Rh(II)-catalysis conditions, **2b** extrudes N_2_ much faster than **1** (15 s vs 8 min to 50% conversion) [14]. The higher reaction rate implies a lower turnover limiting barrier in the catalytic cycle with **2b** compared to **1**. This example made us wonder whether halodiazoacetates have an increased sensitivity (relative to EDA) towards Brønsted and Lewis acids in general. During our previous studies we made the same observations that were originally reported by Schöllkopf [6]. The halodiazoesters readily decompose at room temperature within hours, but they can be handled in solutions at 0 °C or lower temperatures. This significantly reduced thermal stability of halodiazoacetates relative to EDA, in addition to the presumably increased acid sensitivity, inspired us to investigate the properties of the halodiazoacetates in more detail. We therefore set out to study the thermal stabilities of **2a**–**c** by using kinetic data and thus obtaining information of substituent effects.

## Results and Discussion

### Kinetic measurements

**Figure 2:**
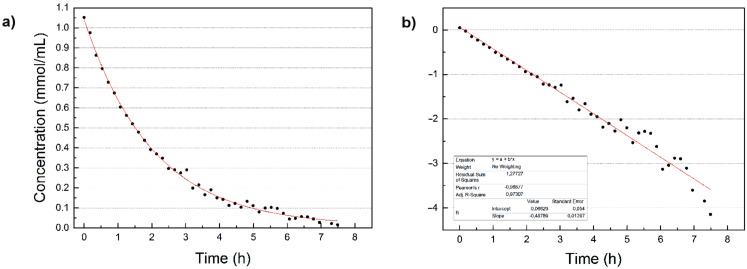
a) The decay of **2b** in toluene-*d*_8_ at 35 °C. b) The plot of log(Δ[**2b**]) vs time.

We synthesized the ethyl halodiazoacetates **2a**–**c** as outlined in Scheme 1 and measured their concentrations vs time in different solvents and at different temperatures and concentrations. The kinetic data for all experiments are presented in Supporting Information File 1 and one representative example is shown in Figure 2.

The data displayed in Figure 2 show an exponential decay of the ethyl bromodiazoacetate (**2b**) concentration which implies first order kinetics. All the kinetic measurements made in this study followed the same first order kinetic profile [15] and the data obtained from the kinetic profiles are summarized in Table 1. As a point of reference, the half-lives (*t*_1/2_) for the non-stabilized diazo compounds 2-diazopropane and 2-diazomethylfurane were reported to be ~3 h at 0 °C [16] and ~2 h at 25 °C [17], respectively. EDA has a reported half-life of 109 h at 100 °C [3], while we measured **2a** to decay with a half-life of 1 h 46 min at 25 °C (Table 1, entry 1).

**Table 1:** Reaction conditions and half-lives (*t*_1/2_) of **2a**–**c**.

Entry	diazo compound **2**	solvent	*T* (°C)	*t*_1/2_	*t*_1/2_ rel.

1	**2a**	toluene-*d*_8_	25	1 h 46 min^a^	0.2
2	**2b**	toluene-*d*_8_	25	7 h 31 min^b^	1.0
3	**2c**	toluene-*d*_8_	25	14 h 59 min^a^	2.0
4	**2b**	toluene-*d*_8_	25	8 h 22 min^c,d^	1.1
5	**2b**	toluene-*d*_8_	25	8 h 29 min^c,e^	1.1
6	**2b**	toluene-*d*_8_	15	16 h 49 min^c^	2.2
7	**2b**	toluene-*d*_8_	35	1 h 25 min^c^	0.2
8	**2b**	CDCl_3_	25	1 h 51 min^c^	0.2
9	**2b**	CD_3_CN	25	3 h 58 min^c^	0.5
10	**2b**	THF-*d*_8_	25	8 h 49 min^c^	1.2
11	**2b**	CD_2_Cl_2_	25	4 h 5 min^c^	0.5
12	**2b**	CD_2_Cl_2_	15	8 h 25 min^c^	1.1
13	**2b**	CD_2_Cl_2_	35	1 h 7 min^c^	0.2
14	**2b**	toluene-*d*_8_	25	10 h 39 min^c,f^	1.4
15	**2b**	CD_2_Cl_2_	25	6 h 45 min^c,g^	0.9
16	**2b**	CDCl_3_	25	6 h 15 min^c,h^	0.8
17	**2b**	CDCl_3_	25	6 h 48 min^c,i^	0.9
18	**2b**	CDCl_3_	25	10 h 54 min^c,j^	1.5
19	**2b**	AcOH	22	63 min^a,k^	0.12
20	**2a**	AcOH	22	33 min^a,k^	0.06
21	**2b**	toluene-*d*_8_	25	23 min^c,l^	0.05

^a^Average of 2 measurements. ^b^Average of 3 measurements. Average concentration 0.57 M. ^c^One measurement. ^d^1.9 M concentration. ^e^3.0 M concentration. ^f^Additive: 20 mol % TMEDA. ^g^Additive: 10 mol % TMEDA. ^h^Additive: 9 mol % TMEDA. ^i^Additive: 14 mol % TMEDA. ^j^Additive: 1 equiv TMEDA. ^k^Glacial acetic acid, [**2**] ~ 0.1 M. ^l^In the presence of <0.03 mol % Cu(OTf)_2_.

The results displayed in Table 1 clearly show that there is a significant difference in the decomposition rate for the three halodiazoacetates **2a**–**c**. The rate of decomposition for **2a** is five times faster than **2b**, while **2c** decomposes two times slower than **2b** (Table 1, entries 1–3). We found the decomposition rate of **2b** to be independent of the initial concentrations; the half-lives were constant (within the experimental error) over the range 0.57 M to 3.0 M (Table 1, entries 2, 4 and 5). As expected, the decomposition rate of **2b** was significantly influenced by temperature (Table 1, entries 2, 6 and 7). Furthermore, the half-life of **2b** was also found to be highly solvent dependent (Table 1, entries 2, 8–11). One obvious reason for the lower stability in CDCl_3_ could be the presence of trace amounts of acid in the used CDCl_3_. However, newly purchased CDCl_3_ that was stored in the fridge over silver foil and 4 Å molecular sieves in order to remove any trace of acid was employed in all experiments. The temperature dependence in toluene-*d*_8_ as solvent was found to be similar in CD_2_Cl_2_ (Table 1, entries 11–13). Next, the effect of additives on the stability of the compounds was investigated. We first tested NEt_3_ (not shown), inspired by the prolonged lifetime of unstabilized diazo compounds in the presence of triethylamine [18], but the additive had no significant effect. The same result was observed in the presence of the metal scavenger Na_2_EDTA. In contrast, tetramethylethylenediamine (TMEDA) as the additive resulted in a significant enhancement of the ethyl bromodiazoacetate lifetime (Table 1, entries 14–18). Thus the half-life of **2b** increased ~6 times in the presence of 1 equiv TMEDA in CDCl_3_ solution. EDA has been reported to be stable in glacial acetic acid [4]. Halodiazoesters **2a** and **2b** on the other hand decomposed with half-lives of approx. 33 min (**2a**) and 63 min (**2b**) in neat acetic acid (Table 1, entries 19 and 20). In the presence of trace amounts of solid Cu(OTf)_2_ in toluene-*d*_8_, **2b** decayed with a half-life of approx. 23 min (Table 1, entry 21). Cu(OTf)_2_ is a well-known catalyst for decomposition of diazo compounds in general [5], but the catalyst loadings are usually two orders of magnitude higher and the reaction times longer in comparison to the decomposition rate of **2b**.

**Scheme 2:**
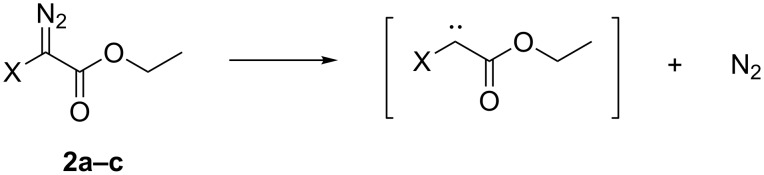
Proposed rate determining step for the thermal decomposition of **2a**–**c**.

There are in principle three pathways for the thermal decomposition of the halo diazoacetates [3]: 1) a unimolecular extrusion of dinitrogen giving N_2_ (g) and a carbene; 2) a bimolecular dimerization leading to azines; and 3) a reaction of the diazo compound with the solvent or a reactant prior to nitrogen extrusion. The fact that the decomposition rate of all halodiazoacetates showed first order kinetics and that *t*_1/2_ for **2b** is independent of its concentration indicates that pathway 1 is most likely. Pathway 1 is illustrated in Scheme 2.

The proposed decomposition pathway is a unimolecular reaction where the rate determining step is the release of N_2_ to form the corresponding free carbene. This is in line with what has been reported for the thermal decomposition of other diazo compounds [3].

### DFT calculations

**Figure 3:**
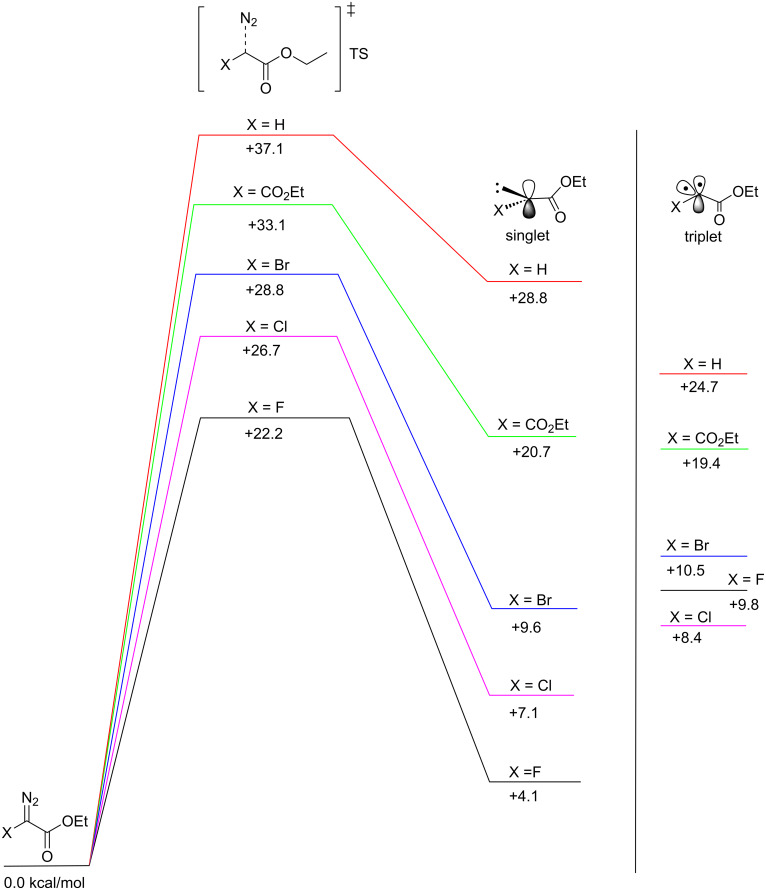
Transition-state energies (kcal/mol) for the release of N_2_ and formation of the singlet carbenes. The corresponding triplet carbenes are displayed to the right.

The proposed unimolecular extrusion of N_2_ to form the free carbene (Scheme 2) was investigated further by DFT calculations for compounds **1** and **2** and the results are summarized in Figure 3. In order to expande the calculation to fluorine, ethyl fluorodiazoacetate (**2d**) was included in the calculations even though the synthesis of this diazo compound has not been reported in the literature. We excluded **2c** in our molecular modeling due to the need for a different basis set for iodine compared to the other halogens. We also included diethyl diazomalonate (**2e**, *t*_1/2_ = 105 h at 100 °C [19]) having an electron-withdrawing α-substituent and allow comparison to literature *t*_1/2_ values. The trends obtained experimentally were confirmed by molecular modeling. EDA has the highest barrier towards loss of N_2_, while the halodiazoacetates follow the order Br > Cl > F. Even though they don’t translate exactly into the *t*_1/2_ values, the heights of the calculated barriers correlate well with the experimentally observed half-lives. The release of N_2_ is an endothermic reaction that produces the free carbene, and according to Hammond’s postulate the transition state is closer in energy to the products than to the reactants. The barrier heights are thus dominated by the energy of the free carbene relative to its diazo precursor. All the studied halodiazoacetates have lower barriers towards N_2_ extrusion compared to EDA because π-donation of a free-electron pair from the halogen to the vacant p-orbital on the carbene carbon affects the electronic structure of the transition states. The halocarboethoxy carbenes are also more stabilized than the carbene generated from EDA. The energy gain from the transition state to the free carbene is 18–20 kcal/mol for X = F, Cl and Br, but only 8 kcal/mol for EDA (Figure 3).

**Figure 4:**
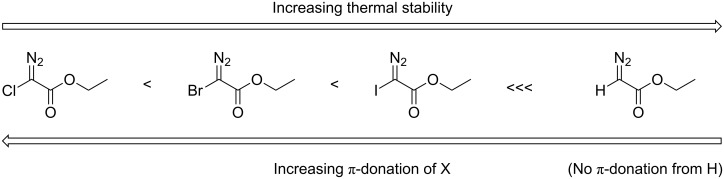
Thermal stability of **1** and **2a**–**c**, and the α-substituents’ contribution to π-donation.

The π-donation ability from the halo substituents to provide carbene stabilization follows the order F > Cl > Br and presumably I as the least stabilizing carbene substituent [20]. The thermal stabilities of ethyl halodiazoacetates and their α-substituent effects are summarized and graphically displayed in Figure 4.

**Figure 5:**
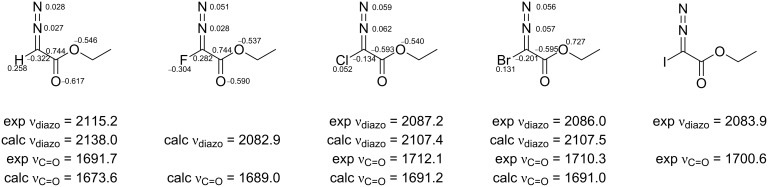
NBO atomic charges and IR stretching frequencies calculated [21] and experimentally recorded for **1** and **2a**–**c**.

We next recorded the IR spectra of **1** and **2a**–**c** and compared their diazo and carbonyl stretching frequencies to the computed frequencies. The results are summarized in Figure 5.

EDA has a significantly higher diazo stretching frequency and lower C=O frequency compared to the halodiazoacetates. Among the halodiazoacetates, there is experimentally a small but steady drop in both the diazo and carbonyl frequencies following the order Cl > Br > I [22]. The DFT calculations also predicted another characteristic trait of the halodiazoacetates: That less nucleophilicity can be inferred from the NBO charges. Based only on NBO charges, halodiazoacetates may be expected to be even less nucleophilic than diethyl diazomalonate (see Supporting Information File 2 for the numbers).

**Figure 6:**
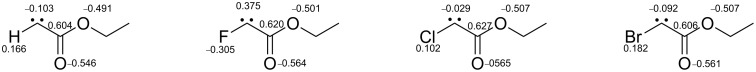
NBO atomic charges of the singlet carbenes from **1** and **2a**,**b**, and **d**.

All of the investigated diazo compounds thermally extrude N_2_ to initially form a singlet carbene with an empty p-orbital at the carbene carbon (see Supporting Information File 2 for bond angles and bond lengths). The NBO atomic charges for the singlet carbenes from **1** and **2a**,**b**, and **d** are shown in Figure 6.

The calculations predict the following order of electrophilicity for the carbenes displayed in Figure 6: F > Cl > Br > H. The atomic charges for the carbenes generated from **2a** and **2d** are fairly similar to the computed values of the closely related halo carbomethoxy carbenes studied by Platz’ research group [23]. We also computed the energies of the corresponding triplet carbenes to obtain an energy difference between the singlet and triplet electronic spin state (Figure 3). The singlet–triplet energy gap is 4.1 kcal/mol in favor of the triplet state for the EDA-generated carbene. All three carbenes generated from **2a**–**c** on the other hand have singlet ground states. The singlet–triplet gap follows the order F > Cl > Br and is small for X = Br (0.9 kcal/mol), slightly larger for X = Cl (1.3 kcal/mol) and rather large (5.7 kcal/mol) for X = F.

### Chemical reactivity

Since the halodiazoacetates decomposed to generate carbenes with a significant rate at room temperature, we briefly investigated the reactivity of the carbenes generated thermally at ambient temperature in a non-catalytic fashion. As a probe, we elected the standard cyclopropanation reaction of styrenes. We found that the presence of styrene and/or methyl 4-nitrobenzoate (internal standard) did not change the rate of the reaction relative to the rate in the absence of the substrates, confirming that the rate limiting step is the release of N_2_ to generate the free carbene. In our initial report on the Rh(II)-catalyzed cyclopropanation high yields were obtained using toluene as solvent [11]. The thermal non-catalytic reaction, however, gave a complex mixture in this solvent. We found dichloromethane to be a more suitable solvent for the thermal reaction and did a back to back comparison to the Rh_2_(esp)_2_-catalyzed reaction in CH_2_Cl_2_. The results are displayed in Table 2 and can be summarized as follows: The thermal decomposition of **2c** gave a complex mixture and only a trace of the cyclopropane could be seen in the ^1^H NMR spectrum of the crude product. The Rh_2_(esp)_2_-catalyzed reactions in CH_2_Cl_2_ gave dramatically lower yields compared to the reactions in toluene [11]. The yields from the thermal reactions were only slightly lower (11–23%) compared to the Rh(II)-catalyzed reactions. The diasteromeric ratios, on the other hand, were significantly higher in the metal-catalyzed reactions.

**Table 2:** Cyclopropanation of styrenes with **2a**–**c**.

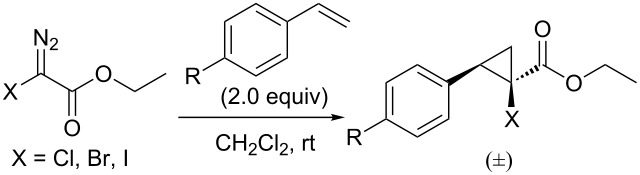						

Entry	X	R	Yield^a,b^	dr^b,c^	Yield^a,d^	dr^c,d^

1	Cl	H	40	4:1	57	10:1
2	Br	H	44	3:1	67	9:1
3	I	H	–	–	44	11:1
4	Cl	OMe	39	3:1	57	7:1
5	Br	OMe	43	2:1	52	10:1
6	Cl	CF3	31	5:1	43	24:1
7	Br	CF3	34	3:1	45	14:1

^a^Isolated yield over two steps from EDA. ^b^Thermal decomposition. ^c^Determined by ^1^H NMR. ^d^In the presence of Rh_2_(esp)_2_ (1 mol %).

## Conclusion

**Figure 7:**
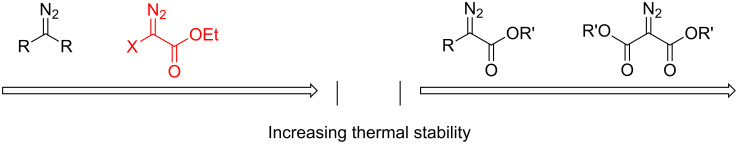
Relative thermal stability of halodiazoacetates (red color).

The thermal stability (or lack thereof) of ethyl halodiazoacetates places them in the same category as the non-stabilized alkyl diazocompounds despite the presence of an α-electron-withdrawing-ester group (Figure 7).

**Figure 8:**
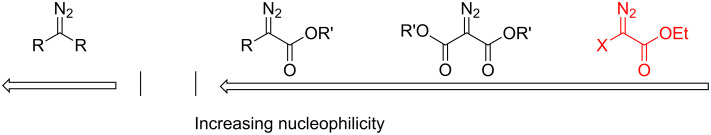
Relative nucleophilicity of halodiazoacetates (red color).

The primary reason for the low stability of halodiazoacetates is the relatively high stability of their corresponding carbenes due to π-donation from the halogens into the carbenes’ vacant p-orbitals. Ethyl halodiazoacetates are also much less stable than EDA in the presence of a weak acid such as acetic acid and dramatically more sensitive towards transition metal salts such as Cu(OTf)_2_ and Rh_2_(II) carboxylates. Calculations predict the diazo carbons in the halodiazoacetates to have a much less nucleophilic character compared to EDA (Figure 8).

The carbenes generated from **2a**–**c** are predicted to be more electrophilic and have singlet ground states compared to the corresponding carbene generated from EDA which has a triplet ground state. The thermally generated carbenes from **2a** and **2b** undergo cyclopropanation reactions of styrenes at room temperature, which are unusually mild conditions for catalyst-free cyclopropanation reactions. All together this puts the ethyl halodiazoacetates into a separate category of diazoacetates where the halodiazoacetates themselves are much more thermally unstable than EDA, while being precursors of stabilized singlet ground-state carbenes which are more electrophilic than the carbene generated from EDA.

## Experimental

### General procedure for the synthesis of halodiazoacetates **2a–c**

EDA (1.0 mmol) was diluted with CH_2_Cl_2_ (10 mL) and the solution was cooled to 0 °C. To this stirring solution was added DBU (1.4 mmol) and stirring was continued at 0 °C for 5 min before the *N*-halosuccinimide (1.1 mmol, NBS or NCS or NIS) of choice was added. There was an immediate color change from yellow to orange or red, and the conversion of EDA was completed in less than 5 min as judged by TLC analysis. After stirring for 5 min at 0 °C, the solution was quickly filtered through a pre-cooled (0 °C) plug (2–3 cm) of silica gel, eluting with cold CH_2_Cl_2_. Compounds **2a–c** were obtained as orange/red solutions in CH_2_Cl_2_ (typically ~1 mmol in 50 mL).

### Kinetic measurements

The concentration of **2a–c** vs time was measured by using ^13^C NMR.

General example: Compound **2b** was prepared according to the general procedure described above. To the solution of **2b** in CH_2_Cl_2_ (0 °C) was added cold (0 °C) toluene-*d*_8_ and dichloromethane was removed in vacuo. To 0.30 mL of the cold solution of **2b** in toluene-*d*_8_ was added 0.20 mL of cold toluene-*d*_8_ containing ethyl 4-nitrobenzoate (0.107 mmol) as internal standard. The concentration of **2b** in the NMR sample was measured and calculated to be 0.61 mmol/mL.

The NMR sample was inserted into the spectrometer with the probe temperature set to 0 °C to minimize decomposition of the ethyl halodiazoacetates. The instrument parameters were then adjusted (tune/match and lock/shim) towards the sample before the probe temperature was raised to the desired temperature and a final shimming was performed before the recordings started.

### Representative procedure for cyclopropanation of styrenes

To a vial with a screw cap was added dry CH_2_Cl_2_ (1 mL), styrene (146 mg, 1.40 mmol, 2.1 equiv), Rh_2_(esp)_2_ (8.5 mg, 0.011 mmol, 0.02 equiv) and a magnetic stirring bar. To the stirred mixture was added a solution of **2b** in CH_2_Cl_2_ (7.00 mL, 0.68 mmol, 1.0 equiv). The vial was capped and left stirring at room temperature for 30 min. The solvent was removed and the crude product was purified by silica gel chromatography (5% EtOAc/hexane) to give 123 mg (0.457 mmol, 67%) of ethyl 1-bromo-2-phenylcyclopropanecarboxylate as a colorless oil.

## Supporting Information

File 1 Detailed experimental procedures and kinetic measurements.

File 2 DFT calculations.
