# Supplementary material for: On the cause of low thermal stability of ethyl halodiazoacetates
Source: Beilstein J Org Chem. 2016 Jul 26;12:1590–7. doi: 10.3762/bjoc.12.155 (PMC4979875; doi:10.3762/bjoc.12.155)
Supplement: File 1 — Detailed experimental procedures and kinetic measurements. [file Beilstein_J_Org_Chem-12-1590-s001.pdf]

Supporting Information File 1

for

On the cause of low thermal stability of ethyl halodiazoacetates

Magnus Mortén, Martin Hennum and Tore Bonge-Hansen*

Address: Department of Chemistry, University of Oslo, P.O. Box 1033 Blindern, NO-0315 Oslo, Norway

Email: Tore Bonge-Hansen* - tore.hansen@kjemi.uio.no

*Corresponding author

Detailed experimental procedures and kinetic measurements

Table of Contents:

General information S2

Calibration of the ^{13}C NMR method used for kinetic measurements S3

Characterization data for halodiazoacetates **2a–c** S4

General procedure for the kinetic measurements using ^{13}C NMR S5

Plots and tables from kinetic experiments S6

Cyclopropanation of styrenes with **2a–c** S15

^{1}H NMR and ^{13}C NMR spectra S24

References S34

General information

All chemicals were used as received from Sigma-Aldrich. NBS and NCS were recrystallized and dried under high vacuum prior to use. Dichloromethane was dried using an MBraun MB-SPS800 solvent purification system. Flash chromatography was performed on an Isco Inc. CombiFlash Companion system with silica gel from Fluka (60 Å, 35–70 µm, high purity, pH 7). Eluents ethyl acetate (EtOAc) and dichloromethane (DCM) of technical grade were used. Thin layer chromatography (TLC) was performed on silica gel coated aluminum sheets from Merck (TLC silica gel, 60 F254). NMR spectra were recorded on a Bruker AVII400 at room temperature at 400 MHz for ^{1}H NMR and 100 MHz for ^{13}C NMR with $CDCl_3$ as solvent, calibrated to 7.24 ppm for ^{1}H NMR and 77.00 ppm for ^{13}C NMR. Chemical shifts (δ) are given in ppm relative to the solvent $CDCl_3$. MS spectra (EI) were recorded on a VG Prospec sector instrument from Fissions Instruments at 70 eV. High resolution mass spectra (EI) were measured on a Micromass Q-Tof-2 mass spectrometer.

All new compounds were fully characterized by NMR as well as GC–MS, EIMS and HRMS–ESI. Previously reported compounds were characterized by ^{1}H NMR and EIMS and compared to the literature data given as references.

Calibration of the ^{13}C NMR method used for kinetic measurements

We initially wanted to use ^{1}H NMR spectroscopy for the kinetic decomposition, not only to measure the decomposition rate of the halo diazoesters but also to analyze the reaction mixture with respect to product formation during and after complete decomposition. However, the ester signals of the halodiazoacetates showed significant overlap with the decomposition products and made quantitative measurements very difficult. ^{13}C NMR is usually not the method of choice for quantitative measurements with accurate integrals, but it can be used when the instrument parameters are carefully adjusted. In order to eliminate the problematic consequences of the NOE effect with respect to signal enhancement, we used an inverse-gated pulse program with the decoupling channel turned off in the time between every pulse. We measured the relaxation constant T_1 (6.8 s in toluene-d_8) for the methyl signal in ethyl diazoacetate (EDA) to give us an approximation for the T_1 value for the halo diazoacetates (Figure S1). In the beginning of this study we used ethyl 4-nitrobenzoate as an internal standard and its T_1 value was determined to be 4.2 seconds. We later used the absolute integrals to measure the decay of the signals of interest.

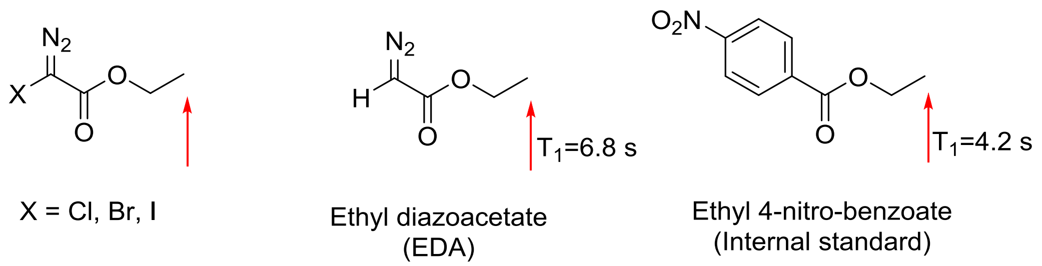

Figure S1. Halodiazoacetates **2a–c**, ethyl diazoacetate (EDA) and ethyl 4-nitrobenzoate (internal standard). The red arrows point to the carbon atoms used for the measurements.

We used a 30° pulse angle and calculated the minimum time delay between pulses to be 0.12 seconds from the Ernst equation. We then used a very conservative estimation of the T_1 values for the halodiazoacetates and set the time delay to 5 seconds in order to avoid saturation of the signals. Then one spectrum (one data point) was recorded every ten minutes from 100 FIDs with a total recording time of 642 seconds for one spectrum. Since **2c** decomposed significantly slower than the Cl and Br-analogs, we put in a time delay between each recording. We found that these parameters gave a satisfactory signal-to-noise (S/N) ratio for concentrations of **2a–c** in the range of 0.50–3.0 mmol/mL. To confirm that the method gave satisfactory integrals over time, we followed the absolute integral of the methyl signal in the internal standard over an eight-hour interval (Figure S2). Since ethyl 4-nitrobenzoate is a stabile compound, the optimum result for the measurements would be a horizontal straight line. As can been seen from Figure S2, there is variation of ca. ±5% from the average value of the measurements. This can be interpreted as an estimate for the error associated with this method. There was no sign of signal saturation since the line is horizontal.

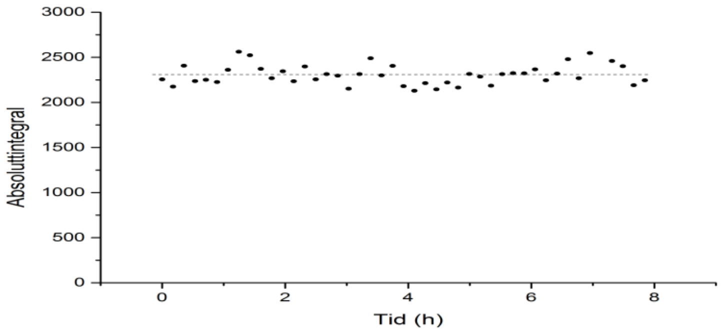

Figure S2. The absolute integral of the methyl signal in ethyl 4-nitrobenzoate over time. The dotted line is the average value for the series.

Characterization data for halodiazoacetates 2a–c

Ethyl bromodiazoacetate (2b)

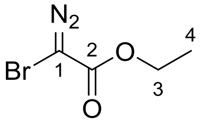

^{1}H-NMR (500 MHz; toluene-d$_8$): δ 0.85 (t, *J* = 7.2 Hz, 3H), 3.83 (q, *J* = 7.2 Hz, 2H).

^{13}C-NMR (125 MHz; toluene-d$_8$): 14.2 (C4), 62.3 (C3), 162.1 (C2).

MS (EI) *m/z* (relative intensity): 192/194 ([M/M+2]$^+$, 3/2), 29 (100).

HR-MS: 191.9531; Calc. for $C_4H_5N_2O_2{}^{79}Br$: 191.9534 (1.7 ppm).

The compound has previously been reported in the literature.[1]

Ethyl chlorodiazoacetate (2a)

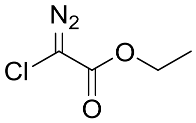

^{1}H-NMR (500 MHz; toluene-d$_8$): δ 0.79-0.87 (m, 3H), 3.79-3.87 (m, 2H).

^{13}C-NMR (125 MHz; toluene-d$_8$): δ 14.2 (C4), 61.9 (C3), 160.9 (C2).

MS (EI) *m/z* (relative intensity): 148 (M$^+$, 1.6%), 29 (100).

The compound has previously been reported in the literature.[1]

Ethyl iododiazoacetate (2c)

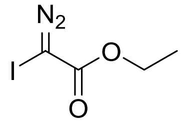

^{1}H-NMR (400 MHz; toluene-d$_8$): δ 0.84 (t, *J* = 7.1 Hz, 3H), 3.83 (q, *J* = 7.1 Hz, 2H)

^{13}C-NMR (400 MHz; toluene-d$_8$): δ 14.2 (C4), 62.5 (C3), 162.9 (C2).

MS (EI) *m/z* (relative intensity): 240 (M^+, 37%), 212 (5), 195 (7), 29 (100).

HR-MS: 239.9402; Calc.for $C_4H_5N_2O_2I$: 239.9396 (-2.5 ppm).

The compound has previously been reported in the literature.[1]

General procedure for the kinetic measurements using ^{13}C NMR

Kinetic measurements

The concentration of **2a–c** vs time was measured by using ^{13}C NMR.

General example: Compound **2b** was prepared according to the general procedure described above. To the solution of **2b** in CH_2Cl_2 (0 °C) was added cold (0 °C) toluene-d_8 and dichloromethane was removed in vacuo. To 0.30 mL of the cold solution of **2b** in toluene-d_8 was added 0.20 mL of cold toluene-d_8 containing ethyl 4-nitrobenzoate (0.107 mmol) as internal standard. The concentration of **2b** in the NMR sample was measured and calculated to be 0.61 mmol/mL.

NMR-recordings and data processing

The NMR sample was inserted into the spectrometer with the probe temperature set to 0 °C to minimize decomposition of the ethyl halodiazoacetates. The instrument parameters were then adjusted (tune/match and lock/shim) towards the sample before the probe temperature was raised to the desired temperature and a final shimming was performed before the recordings started. A summary of the most important spectrometer parameters is displayed in the Table S1 below.

Table S1. Key ^{13}C NMR parameters used.

**Spectrometer**	Bruker DRX 125 MHz
**Probe**	5 mm TXI ($^{1}H/^{13}C,^{15}N-^{2}H$) triple resonance invers probe
**Pulse program**	C13IG
**Pulse angle**	30°
**# of FIDs**	100
**d1**	5 sec
**at[a]**	1.09 sec
**Total recordingtime**	642 sec

[a]FID recording time

MestReNova (8.0.1) was used to process the NMR spectra. The concentrations of **2a–c** at $t = 0$ were either calculated relative to the internal standard or calculated based on the amount of EDA used in the preparation of the halodiazoacetates and addition of a carefully measured amount of deuterated solvent. All other concentrations of **2a–c** were then measured relative to the starting concentration at $t = 0$ and plotted against time. Below are tables for the different measurements and one representative plot for each kind of experiment.

Decay plots from the kinetic experiments and the straight lines obtained by plotting log(Δ[2a–c]) vs time.

Compound 2b in toluene-d_8 at 25 °C.

Table S2. Rate constants and half-lives for **2b** in toluene-d_8 at 25 °C.

Sample #	Concentration (mmol/mL)	k (h^{-1})	Half-life
1	0.61	0.0837	8 h 17 min
2	0.49	0.0810	8 h 33 min
3	0.61	0.112	6 h 11 min
**Average**	**0.57**	**0.0923**	**7 h 31 min**

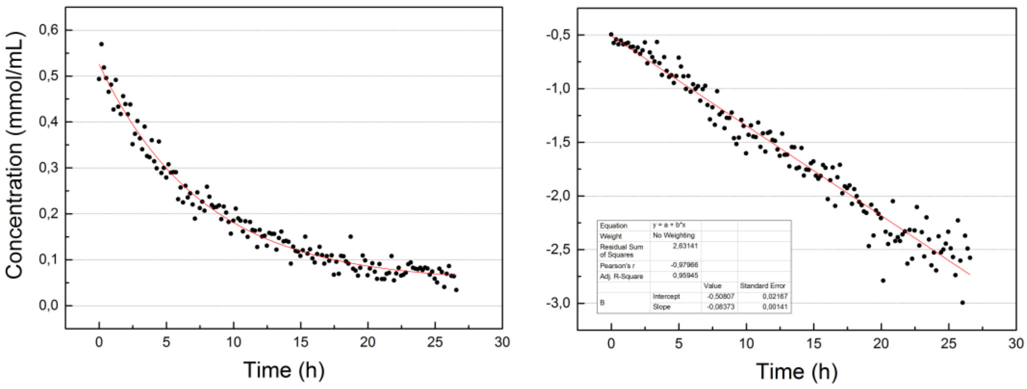

Figure S3. The decay of **2b** in toluene-d_8 at 25 °C over time (left) and the linear plot of log(Δ[**2b**]) vs time (right).

Compound 2a in toluene-*d*$_8$ at 25 °C.

Table S3. Rate constants and half-lives for **2a** in toluene-d_8 at 25 °C.

Sample #	Concentration (mmol/mL)	k (h^{-1})	Half-life
1	0.43	0.419	1h 39 min
2	0.47	0.363	1h 55 min
**Average**	**0.45**	**0.391**	**1 h 46 min**

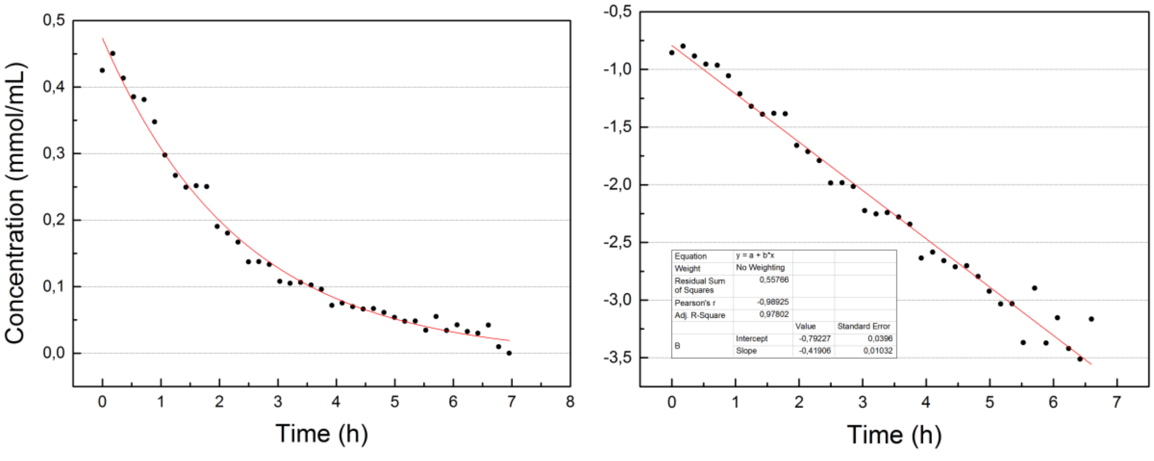

Figure S4. The decay of **2a** in toluene-d_8 at 25 °C over time (left) and the linear plot of log(Δ[**2a**]) vs time (right).

Compound 2c in toluene-d_8 at 25 °C.

Table S4. Rate constants and half-lives for **2c** in toluene-d_8 at 25 °C.

Sample #	Concentration (mmol/mL)	k (h^{-1})	Half-life
1	0.90	0.0520	13 h 20 min
2	0.91	0.0405	17 h 7min
**Average**	**0.91**	**0.0463**	**14h 59 min**

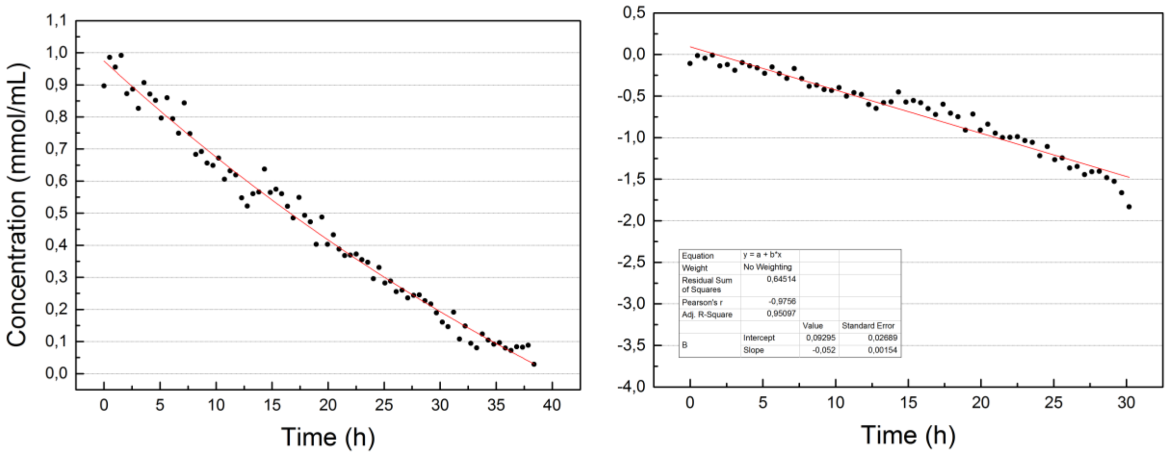

Figure S5. The decay of **2c** in toluene-d_8 at 25 °C over time (left) and the linear plot log(Δ[**2c**]) vs time(right).

Compound 2b in toluene-d_8 at 25 °C, **concentration dependence**.

Table S5. Rate constant and half-life for **2b** in toluene-d_8 at 25 °C and 1.9 M concentration.

Sample #	Concentration (mmol/mL)	k (h^{-1})	Half-life
1	1.9	0.0828	8 h 22 min

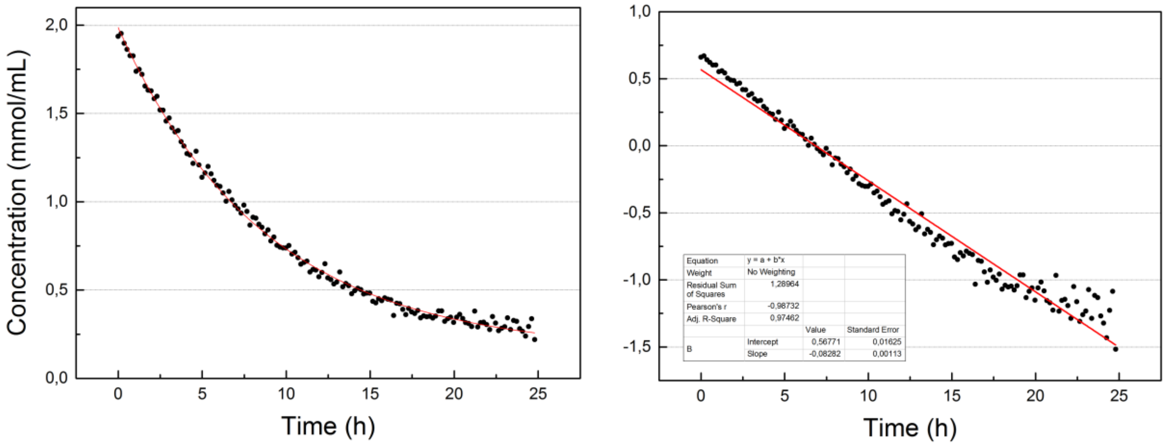

Figure S6. The decay of **2b** in toluene-d_8 at 25 °C over time (left) and its linear plot log(Δ[**2b**]) vs time (right).

Table S6. Rate constant and half-life for **2b** in toluene-d_8 at 25 °C and 3.0 M concentration.

Sample #	Concentration (mmol/mL)	k (h^{-1})	Half-life
1	3.0	0.0818	8 h 29 min

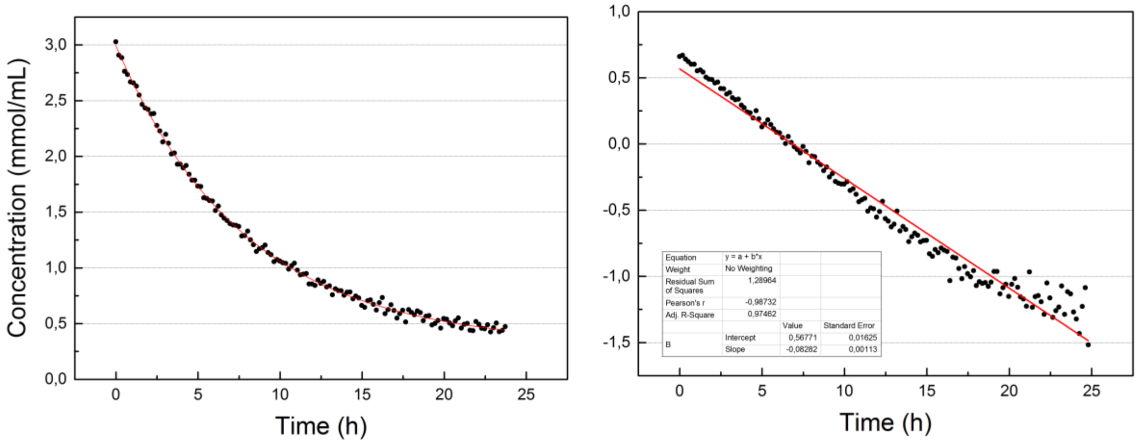

Figure S7. The decay of **2b** in toluene-d_8 at 25 °C over time (left) and its linear plot log(Δ[**2b**]) vs time (right).

Compound 2b in toluene-d_8, temperature dependence.

Table S7. Rate constant and half-life for **2b** in toluene-d_8 at 15 °C.

Sample #	Concentration (mmol/mL)	Temperature (°C)	k (h^{-1})	Half-life
1	1.1	15	0.0412	16 h 49 min

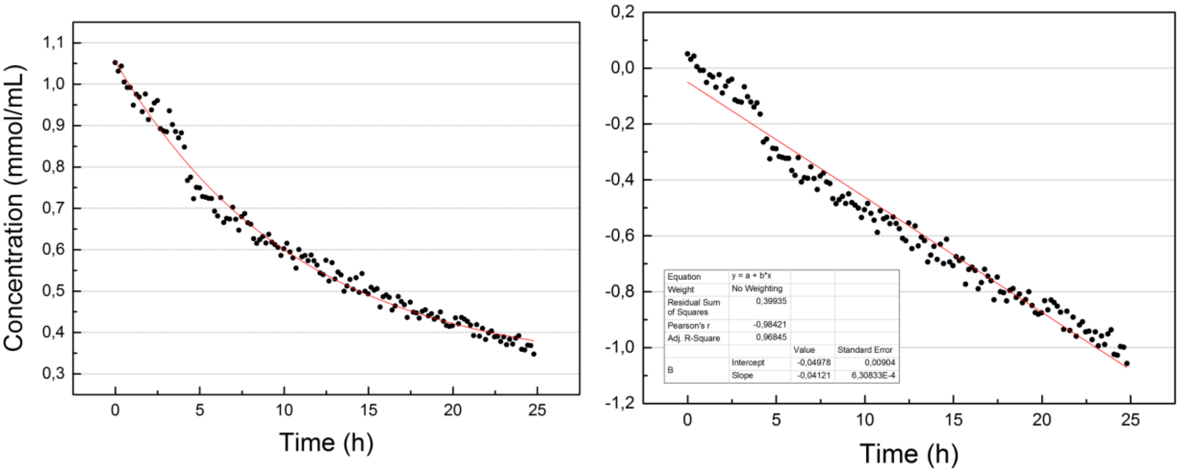

Figure S8. The decay of **2b** in toluene-d_8 at 15 °C over time (left) and its linear plot log(Δ[**2b**]) vs time (right).

Table S8. Rate constant and half-life for **2b** in toluene-d_8 at 35 °C.

Sample #	Concentration (mmol/mL)	Temperature (°C)	k (h^{-1})	Half-life
1	1.1	35	0.488	1h 25 min

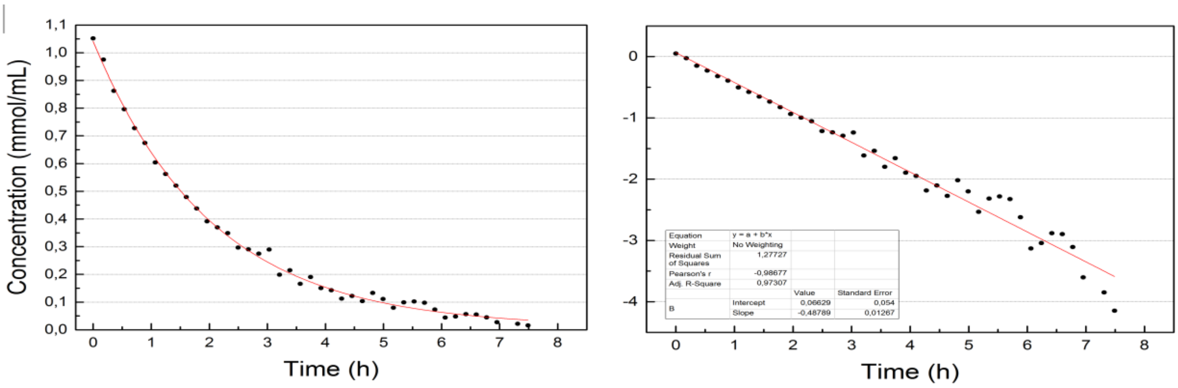

Figure S9. The decay of **2b** in toluene-d_8 at 15 °C over time (left) and its linear plot log(Δ[**2b**]) vs time (right).

Compound 2b in dichloromethane-d_2, temperature dependence.

Table S9. Rate constant and half-life for **2b** in dichloromethane-d_2 at 25 °C.

Sample #	Concentration (mmol/mL)	k (h^{-1})	Half-life
1	0.73	0.170	4 h 5 min

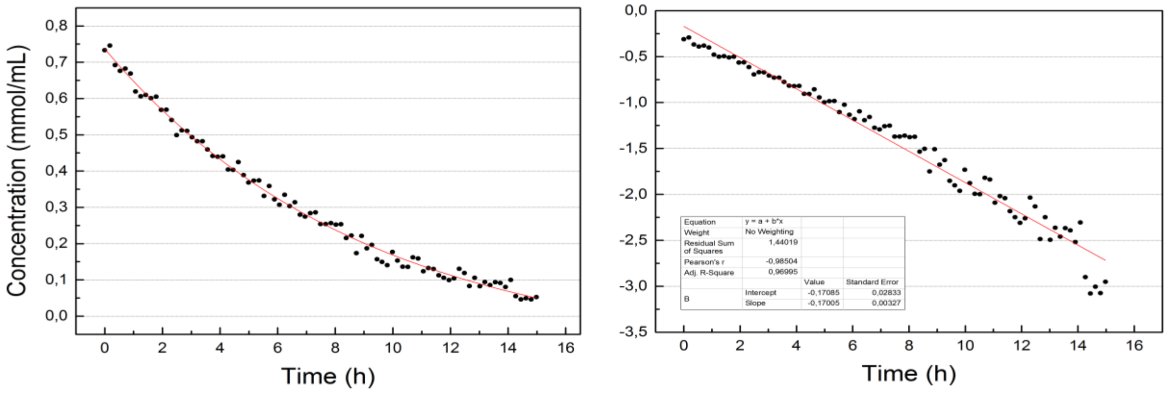

Figure S10. The decay of **2b** in CD_2Cl_2 at 25 °C over time (left) and its linear plot log(Δ[**2b**]) vs time (right).

Table S10. Rate constant and half-life for **2b** in dichloromethane-d_2 at 15 °C.

Sample #	Concentration (mmol/mL)	Temperature (°C)	k (h^{-1})	Half-life
1	0.73	15	0.0823	8 h 25 min

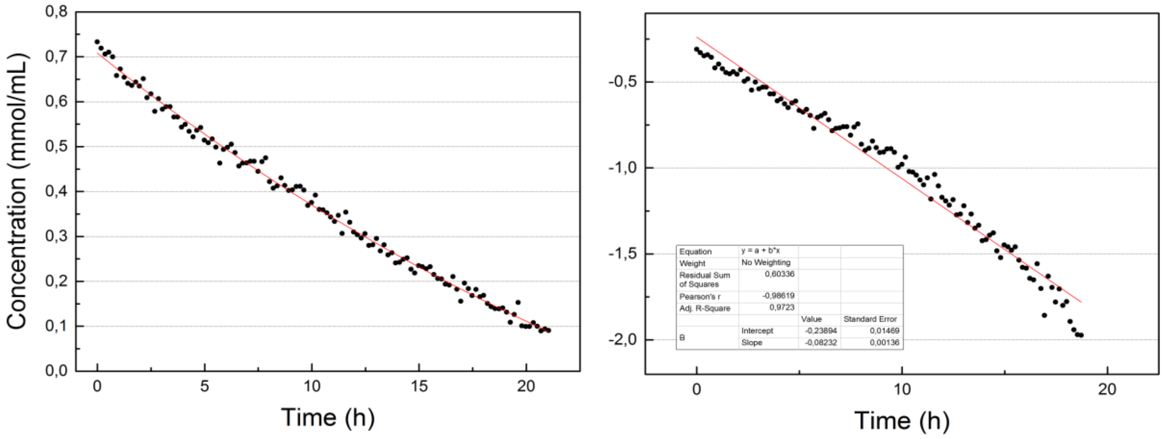

Figure S11. The decay of **2b** in CD_2Cl_2 at 15 °C over time (left) and its linear plot log(Δ[**2b**]) vs time (right).

Table S11. Rate constant and half-life for **2b** in dichloromethane-d_2 at 35 °C.

Sample #	Concentration (mmol/mL)	Temperature (°C)	k (h^{-1})	Half-life
1	0.73	35	0.624	1h 7 min

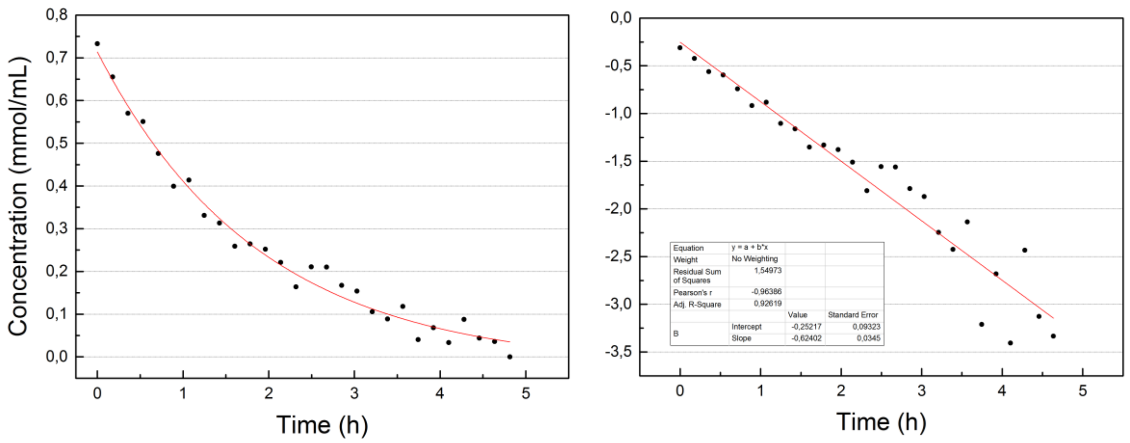

Figure S12. The decay of **2b** in CD_2Cl_2 at 35 °C over time (left) and its linear plot log(Δ[**2b**]) vs time (right).

Table S12. Rate constant and half-life for **2b** in acetonitrile-d_3 at 25 °C.

Sample #	Concentration (mmol/mL)	k (h^{-1})	Half-life
1	0.83	0.175	3h 58 min

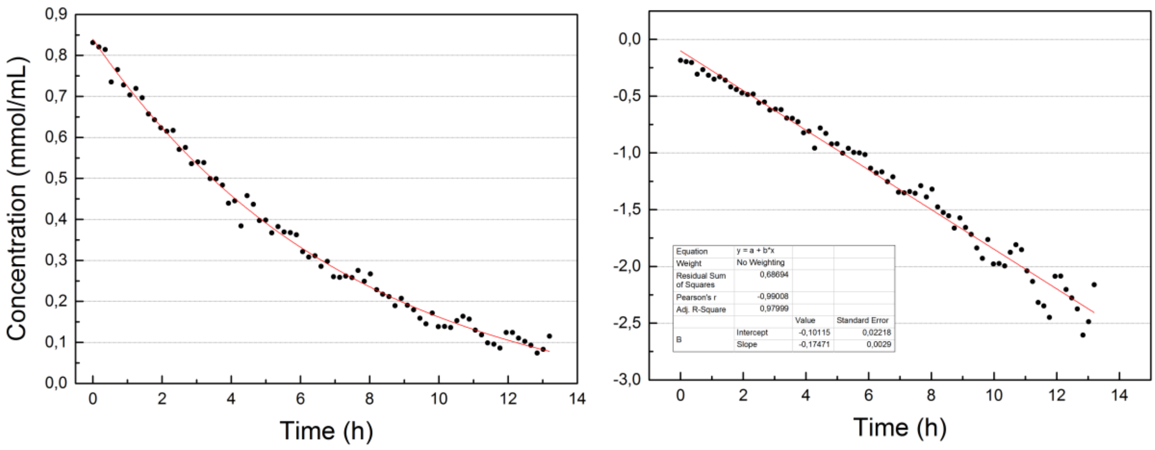

Figure S13. The decay of **2b** in CD_3CN at 25 °C over time (left) and its linear plot log(Δ[**2b**]) vs time (right).

Table S13. Rate constant and half-life for **2b** in THF-d_8.

Sample #	Concentration (mmol/mL)	k (h^{-1})	Half-life
1	0.89	0.0787	8 h 49 min

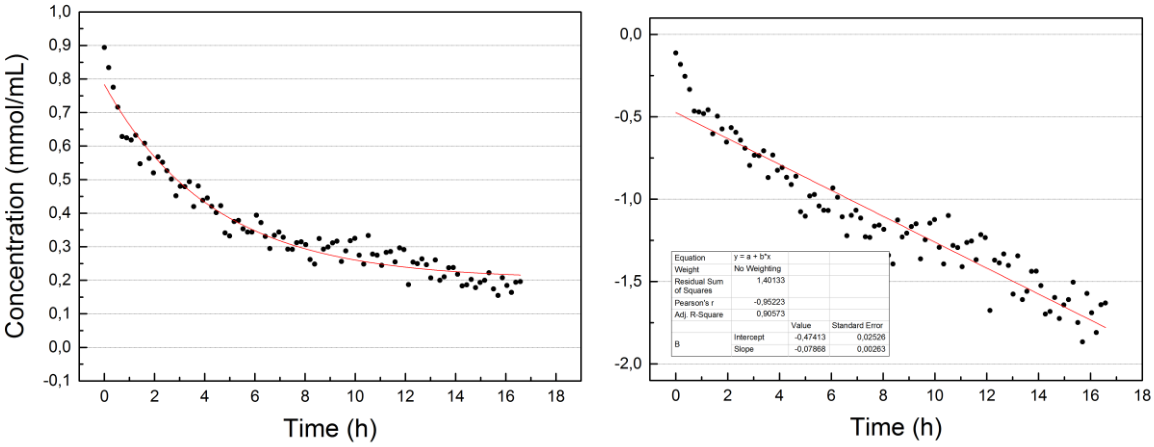

Figure S14. The decay of **2b** in THF-d_8 at 25 °C over time (left) and its linear plot log(Δ[**2b**]) vs time (right).

Table S14. Rate constant and half-life for **2b** in $CDCl_3$ at 25 °C.

Sample #	Concentration (mmol/mL)	k (h^{-1})	Half-life
1	0.76	0.375	1h 51 min

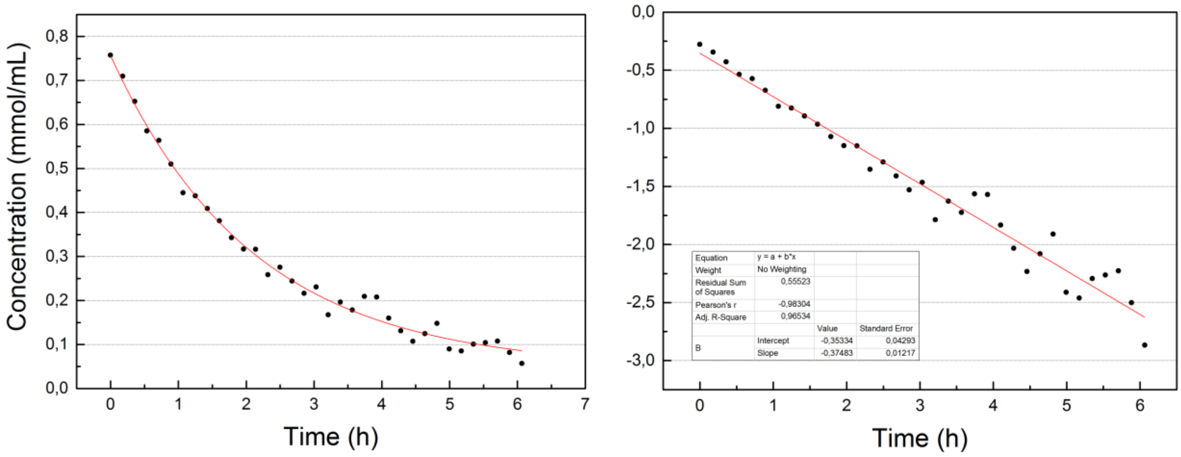

Figure S15. The decay of **2b** in $CDCl_3$ at 25 °C over time (left) and its linear plot log(Δ[**2b**]) vs time (right).

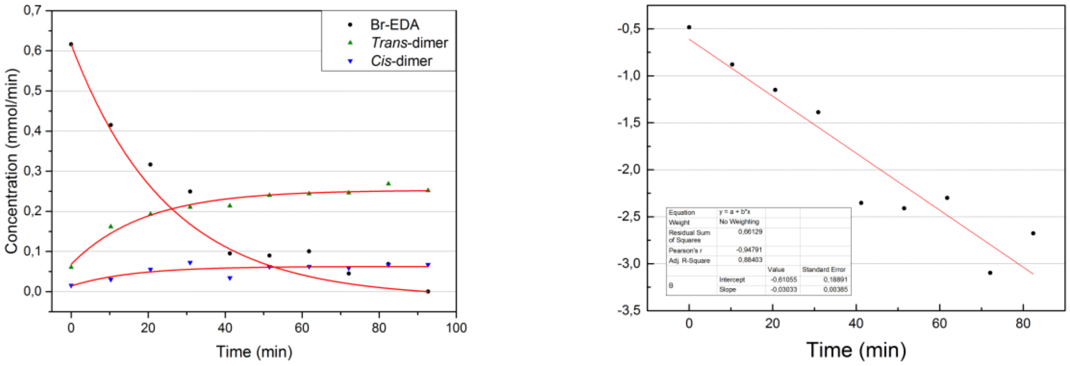

Figure S16. The decay of **2b** in $CDCl_3$ at 25 °C with < 0.03 mol % $Cu(OTf)_2$ over time (left) and its linear plot log(Δ[**2b**]) vs time (right).

The decay of **2a** in AcOH was measured by volumetric release of N_2.

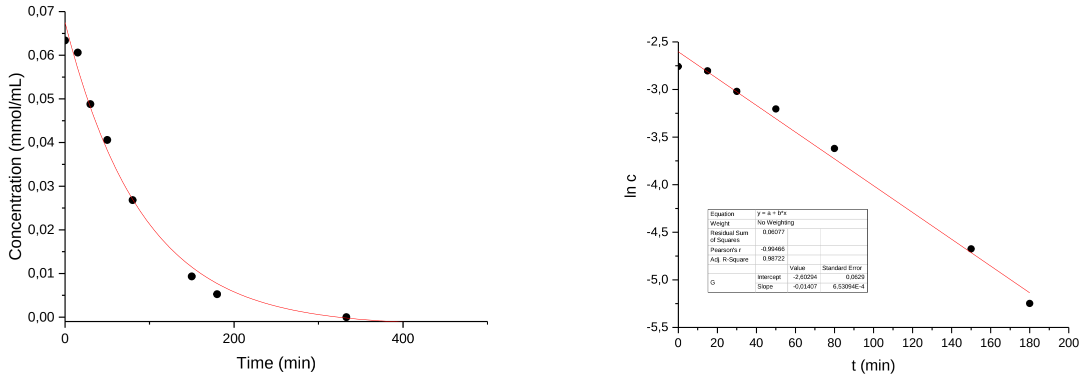

Figure S17. The decay of **2a** in AcOH at 23 °C over time (left) and its linear plot log(Δ[**2a**]) vs time (right).

The decay of **2b** in AcOH was measured by volumetric release of N_2.

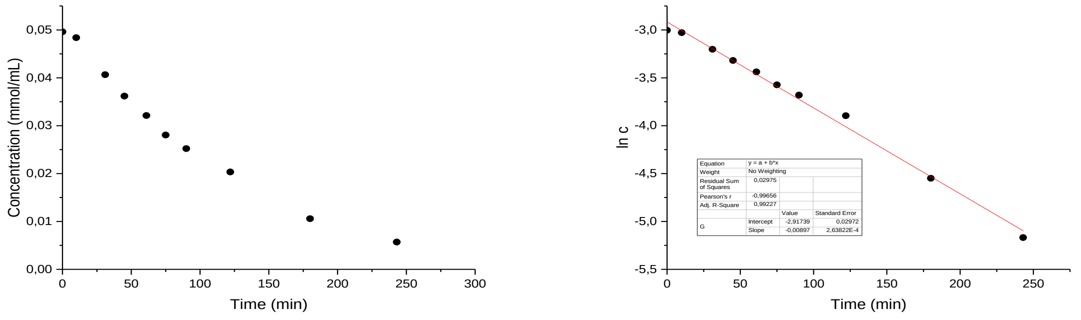

Figure S18. The decay of **2b** in AcOH at 23 °C over time (left) and its linear plot log(Δ[**2b**]) vs time (right).

Cyclopropanation of styrenes with ethyl halodiazoacetates 2a–c

General information.

Halodiazoacetates **2a–c** were synthesized from EDA as described above using the previously published procedure.[1] All cyclopropanes were isolated as a mixture of two diastereoisomers and the reported yields are for the combined mass of the two diastereomers. The product mixtures were analyzed by GC–MS, EIMS, HRMS, ^{1}H NMR, ^{13}C NMR, DEPT135, TOCSY, HSQC and HMBC. The major diastereomer has the aromatic ring and the ester moiety in a *trans* relationship on the cyclopropane ring.

Representative procedure catalytic conditions.

To a vial with a screw cap was added dry CH_2Cl_2 (1 mL), styrene (146 mg, 1.40 mmol, 2.1 equiv), $Rh_2(esp)_2$ (8.5 mg, 0.011 mmol, 0.02 equiv) and a magnetic stirring bar. To this stirring mixture was added a solution of **2b** in CH_2Cl_2 (7.00 mL, 0.680 mmol, 1.0 equiv). The vial was capped and left stirring at room temperature for 30 min. The solvent was removed (rotavap) and the crude product was purified by silica gel chromatography (5% EtOAc/hexane) to give 123 mg (0.457 mmol, 67%) of ethyl 1-bromo-2-phenylcyclopropanecarboxylate as a colorless oil.

Representative procedure thermal, non-catalytic conditions.

To a vial with a screw cap was added dry CH_2Cl_2 (1 mL), styrene (146 mg, 1.40 mmol, 2.1 equiv) and a magnetic stirring bar. To this stirring mixture was added a solution of **2b** in CH_2Cl_2 (7.00 mL, 0.680 mmol, 1.0 equiv). The vial was capped, covered with aluminum foil and left stirring at room temperature for 24 h or until complete consumption of **2b** as judged by TLC analysis. The solvent was removed (rotavap) and the crude product was purified by silica gel chromatography (5% EtOAc/hexane) to give 81.0 mg (0.301 mmol, 44%) of ethyl 1-bromo-2-phenylcyclopropanecarboxylate as a colorless oil.

Ethyl 1-bromo-2-phenylcyclopropane-1-carboxylate

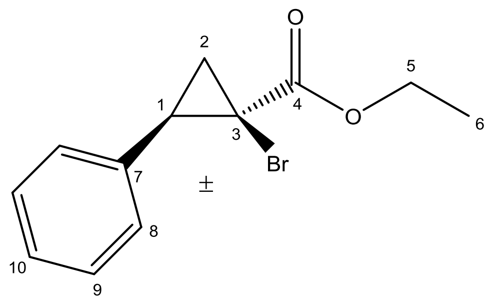

Catalytic conditions: **2b** (0.680 mmol, 1.0 equiv), styrene (146 mg, 1.40 mmol, 2.1 equiv) and $Rh_2(esp)_2$ (8.5 mg, 0.011 mmol, 0.02 equiv) gave 123 mg (0.457 mmol, 67%, dr 9:1) of ethyl 1-bromo-2-phenylcyclopropane-1-carboxylate as a colorless oil.

Thermal conditions: **2b** (0.680 mmol, 1.0 equiv) and styrene (146 mg, 1.40 mmol, 2.1 equiv) gave 81.0 mg (0.301 mmol, 44%, dr 3:1) of ethyl 1-bromo-2-phenylcyclopropane-1-carboxylate as a colorless oil.

Major diastereomer (structure displayed in drawing):

^{1}H-NMR (600 MHz; $CDCl_3$): δ 1.32 (t, *J* = 7.1 Hz, 3H, H6), 1.78 (dd, *J* = 8.5, 6.0 Hz, 1H, H2), 2.19 (dd, *J* = 10.2, 6.0 Hz, 1H, H2), 2.91-2.97 (m, 1H, H1), 4.23-4.28 (m, 2H, H5), 7.22-7.35 (m, 5H, H-Ar).

^{13}**C-NMR** (150 MHz; $CDCl_3$): δ 14.1 (C6), 23.9 (C2), 33.2 (C1), 35.3 (C3), 62.8 (C5), 127.5 (C-Ar), 128.1 (C-Ar), 129.4 (C-Ar), 135.7 (C-Ar), 169.9 (C4).

GC-MS (R_t, *m/z*): 23 min, 268/270 ($[M/M+2]^+$).

Minor diastereomer (structure not displayed):

^{1}**H-NMR** (600 MHz; $CDCl_3$): δ 0.85 (t, *J* = 7.1 Hz, 3H, H6), 1.75-1.76 (m, 1H, H2), 2.35 (dd, *J* = 8.4, 6.6 Hz, 1H, H2), 3.06 (dd, *J* = 10.0, 8.4 Hz, 1H, H1), 3.77-3.91 (m, 2H, H5), 7.22-7.35 (m, 5H, H-Ar).

^{13}**C-NMR** (150 MHz; $CDCl_3$): δ 13.6 (C6), 21.9 (C2), 31.7 (C1), 36.6 (C3), 62.1 (C5), 127.3 (C-Ar), 128.1 (C-Ar), 128.8 (C-Ar), 134.4 (C-Ar), 166.9 (C4).

GC-MS (R_t, *m/z*): 22 min, 268/270 ($[M/M+2]^+$).

MS (EI) *m/z* (relative intensity): 268/270 ($[M/M+2]^+$, 22/22), 239 (3), 189 (5), 116 (35), 115 (100).

HR-MS: 268.0095; calc for $C_{12}H_{13}{}^{79}BrO_2$: 268.0099 (1.3 ppm).

This compound has previously been reported in the literature.[1]

Ethyl 1-chloro-2-phenylcyclopropane-1-carboxylate

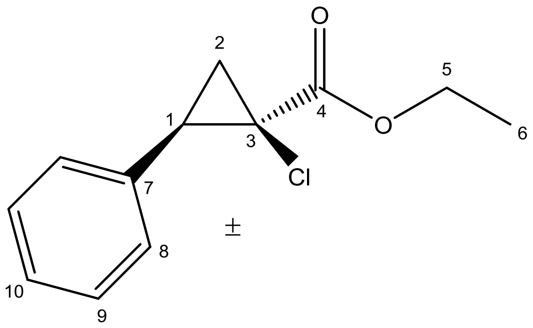

Catalytic: **2a** (0.665 mmol, 1.0 equiv), styrene (139 mg, 1.33 mmol, 2.0 equiv) and $Rh_2(esp)_2$ (8.0 mg, 0.011 mmol, 0.02 equiv) gave 83.5 mg (0.370 mmol, 57%, dr 10:1) of ethyl 1-chloro-2-phenylcyclopropane-1-carboxylate as a colorless oil.

Thermal: **2a** (0.665 mmol, 1.0 equiv) and styrene (139 mg, 1.33 mmol, 2.0 equiv) gave 60.1 mg (0.267 mmol, 40%, dr 4:1) of ethyl 1-chloro-2-phenylcyclopropane-1-carboxylate as a colorless oil.

Major diastereomer (structure displayed in drawing):

^{1}H-NMR (600 MHz; $CDCl_3$): δ 1.32 (t, *J* = 7.1 Hz, 3H, H6), 1.74 (dd, *J* = 8.5, 6.0 Hz, 1H, H2), 2.13 (dd, *J* = 10.2, 6.0 Hz, 1H, H2), 3.04-3.08 (m, 1H, H1), 4.26 (dq, *J* = 7.1, 1.3 Hz, 2H, H5), δ 7.21-7.36 (m, 5H, H-Ar).

^{13}C-NMR (150 MHz; $CDCl_3$): δ 14.1 (C6), 23.5 (C3), 33.7 (C1), 44.8 (C3), 62.6 (C5), 127.5 (C-Ar), 128.2 (C-Ar), 129.4 (C-Ar), 134.5 (C-Ar), 170.2 (C4).

GC-MS (R_t, *m/z*): 22 min, 224 (M^+).

Minor diastereomer (structure not displayed):

^{1}H-NMR (600 MHz; $CDCl_3$): δ 0.84-0.88 (m, 3H, H6), 1.75-1.77 (m, 1H, H2), 2.32 (dd, *J* = 8.6, 6.4 Hz, 1H, H2), 2.99-3.02 (m, 1H, H1), 3.78-3.91 (m, 2H, H5), 7.19-7.37 (m, 5H, H-Ar).

^{13}C-NMR (150 MHz; $CDCl_3$): δ 13.6 (C6), 21.8 (C3), 31.6 (C1), 36.8 (C3), 61.9 (C5), 127.4 (C-Ar), 128.1 (C-Ar), 128.9 (C-Ar), 134.4(C-Ar), 167.3 (C4).

GC-MS (R_t, *m/z*): 21 min, 224 (M^+).

MS (EI) *m/z* (relative intensity): 224/226 (M^+, 40/14), 195 (9), 151 (12), 116 (31), 115 (100).

HR-MS: 224.0607; calc for $C_{12}H_{13}ClO_2$: 224.0604 (-1.5 ppm).

This compound has previously been reported in the literature.[1]

Ethyl 1-iodo-2-phenylcyclopropane-1-carboxylate

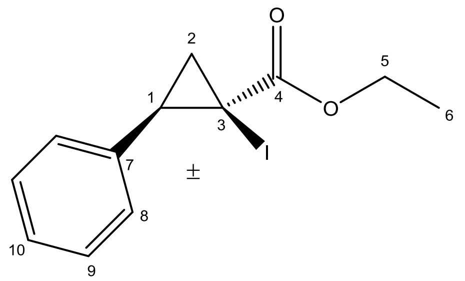

Catalytic: **2c** (0.662 mmol, 1.0 equiv), styrene (138 mg, 1.33 mmol, 2.0 equiv) and $Rh_2(esp)_2$ (8.2 mg, 0.011 mmol, 0.02 equiv) gave 92.1 mg (0.291 mmol, 44 %, 11:1) of ethyl-1-iodo-2-phenylcyclopropane-1-carboxylate as a colorless oil.

Thermal: Only trace of the product was detected in the crude ^{1}H NMR spectrum.

Major diastereomer (structure displayed in drawing):

^{1}H-NMR (600 MHz; $CDCl_3$): δ 1.31 (t, *J* = 7.1 Hz, 3H, H6), 1.72 (dd, *J* = 8.3, 5.9 Hz, 1H, H2), 2.27 (dd, *J* = 9.9, 5.9 Hz, 1H, H2), 2.54-2.57 (m, 1H, H1), 4.21 (dq, *J* = 7.2, 1.3 Hz, 2H, H5), 7.14-7.37 (m, 5H, H-Ar).

^{13}C-NMR (150 MHz; $CDCl_3$): δ 9.5 (C6), 14.1 (C2), 25.2 (C1), 33.1 (C3), 63.0 (C5), 127.6 (C-Ar), 128.1 (C-Ar), 129.3 (C-Ar), 138.0 (C-Ar), 170.0 (C4).

GC-MS (R_t, *m/z*): 24 min, 316 (M^+).

Minor diastereomer (structure not displayed):

^{1}H-NMR (600 MHz; $CDCl_3$): δ 0.82 (t, *J* = 7.1 Hz, 3H, H6). 1.63 (dd, *J* = 9.5, 6.7 Hz, 1H, H2), 2.34 (dd, *J* = 8.0, 6.7 Hz, 1H, H2), 2.95 (dd, *J* = 9.5, 7.8 Hz, 1H), 3.74-3.86 (m, 2H, H5), δ 7.15-7.38 (m, 5H, H-Ar).

^{13}C-NMR (150 MHz; $CDCl_3$): δ 13.6 (C6), 13.9 (C2), 22.8 (C1), 37.2 (C3), 62.1 (C5), 127.3 (C-Ar), 128.1 (C-Ar), 128.7 (C-Ar), 133.8 (C-Ar), 168.8 (C4).

GC-MS (R_t, *m/z*): 23 min, 316 (M^+).

MS (EI) m/z (relative intensity): 316 (M^+, 64%), 287 (3), 271 (5), 160 (24), 116 (40), 115 (100).

HR-MS: 315.9956; calc for $C_{12}H_{13}IO_2$: 315.9960 (1.3 ppm).

This compound has previously been reported in the literature.[1]

Ethyl 1-bromo-2-(4-methoxyphenyl)cyclopropane-1-carboxylate

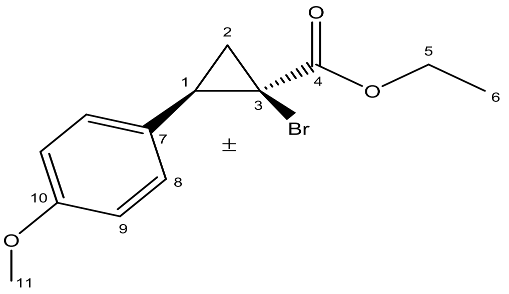

Catalytic: **2b** (0.597 mmol, 1.0 equiv), 4-methoxystyrene (160 mg, 1.19 mmol, 2.0 equiv) and $Rh_2(esp)_2$ (6.3 mg, 0.0083 mmol, 0.01 equiv) gave 92.6 mg (0.310 mmol, 52%, dr 10:1) of ethyl 1-bromo-2-(4-methoxyphenyl)cyclopropane-1-carboxylate as a colorless oil.

Thermal: **2b** (0.597 mmol, 1.0 equiv) and 4-methoxystyrene (160 mg, 1.19 mmol, 2.0 equiv) gave 76.7 mg (0.256 mmol, 43%, dr 2:1) of ethyl 1-bromo-2-(4-methoxyphenyl)cyclopropane-1-carboxylate as a colorless oil.

Major diastereomer (structure displayed in drawing):

^{1}H-NMR (600 MHz; $CDCl_3$): δ 1.31 (t, *J* = 7.1 Hz, 3H, H6), 1.71 (dd, *J* = 8.5, 6.0 Hz, 1H, H2), 2.17 (dd, *J* = 10.2, 6.0 Hz, 1H, H2), 2.84-2.90 (m, 1H, H1), 3.79 (s, 3H, H11), 4.24 (dq, *J* = 7.1, 2.0 Hz, 2H, H5), 6.83-6.89 (m, 2H, H-Ar), 7.10-7.17 (m, 2H, H-Ar).

^{13}C-NMR (150 MHz; $CDCl_3$): δ 14.1 (C6), 23.6 (C2), 33.1 (C1), 35.8 (C3), 55.2 (C11), 62.9 (C5), 113.5 (C-Ar), 129.8 (C-Ar), 130.5 (C-Ar), 159.0 (C-Ar), 169.8 (C4).

GC-MS (R_t, *m/z*): 26 min, 298/300 ($[M/M+2]^+$).

Minor diastereomer (structure not displayed):

^{1}H-NMR (600 MHz; $CDCl_3$): δ 0.91 (t, *J* = 7.1 Hz, 3H, H6), 1.71-1.74 (m, 1H, H2), 2.30 (dd, *J* = 8.4, 6.6 Hz, 1H, H2), 2.96-3.02 (m, 1H, H1), 3.75 (s, 3H, H7), 3.81-3.93 (m, 2H, H5), 6.78 (d, *J* = 8.7 Hz, 2H, H-Ar), 7.10-7.17 (m, 2H, H-Ar).

^{13}C-NMR (150 MHz; $CDCl_3$): δ 14.0 (C6), 24.1 (C2), 32.8 (C1), 36.1 (C3), 55.2 (C11), 62.7 (C5), 113.5 (C-Ar), 127.8 (C-Ar), 130.5 (C-Ar), 158.8 (C-Ar), 170.0 (C4).

GC-MS (R_t, *m/z*): 25 min, 298/300 ($[M/M+2]^+$).

MS (EI) *m/z* (relative intensity): 298/300 ($[M/M+2]^+$, 16/16), 269/271 (11/11), 219 (100), 154 (88).

HR-MS: 298.0209; calc for $C_{13}H_{15}O_3{}^{79}Br$: 298.0205 (-1.5 ppm)

This compound has previously been reported in the literature.[1]

Ethyl 1-chloro-2-(4-methoxyphenyl)cyclopropane-1-carboxylate

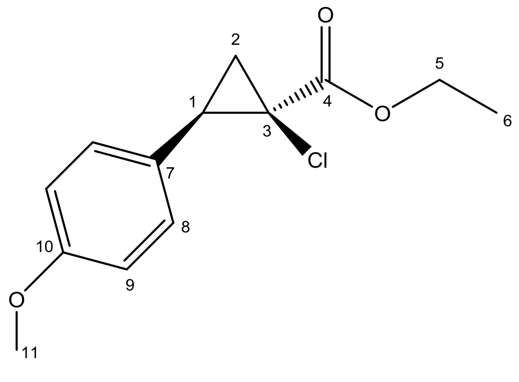

Catalytic: **2a** (0.597 mmol, 1.0 equiv), 4-methoxystyrene (160 mg, 1.19 mmol, 2.0 equiv) and $Rh_2(esp)_2$ (7.6 mg, 0.010 mmol, 0.02 equiv) gave 86.7 mg (0.340 mmol, 57%, dr 7:1) of ethyl 1-chloro-2-(4-methoxyphenyl)cyclopropane-1-carboxylate as a colorless oil.

Thermal: **2a** (0.597 mmol, 1.0 equiv) and 4-methoxystyrene (160 mg, 1.19 mmol, 2.0 equiv) gave 58.6 mg (0.230 mmol, 39%, dr 3:1) of ethyl 1-chloro-2-(4-methoxyphenyl)cyclopropane-1-carboxylate as a colorless oil.

Major diastereomer (structure displayed in drawing):

^{1}H-NMR (600 MHz; $CDCl_3$): δ 1.32 (t, *J* = 7.2 Hz, 3H, H6), 1.67 (dd, *J* = 8.5, 5.9 Hz, 1H, H2), 2.11 (dd, *J* = 10.2, 5.9 Hz, 1H, H2), 2.97-3.02 (m, 1H, H1), 3.79 (s, 3H, H11), 4.25 (dq, *J* = 7.2, 1.6 Hz, 2H, H5), 6.84-6.89 (m, 2H, H-Ar), 7.12-7.17 (m, 2H, H-Ar).

^{13}C-NMR (150 MHz; $CDCl_3$): δ 14.1 (C6), 23.6 (C2), 33.2 (C1), 45.0 (C3), 55.2 (C11), 62.3 (C5), 113.6 (C-Ar), 126.5 (C-Ar), 130.5 (C-Ar), 159.0 (C-Ar), 170.3 (C4).

GC-MS (R_t, *m/z*): 25 min, 254 (M^+).

Minor diastereomer (structure not displayed):

^{1}H-NMR (600 MHz; $CDCl_3$): δ 0.92 (t, *J* = 7.1 Hz, 3H, H6), 1.72 (dd, *J* = 10.1, 6.4 Hz, 1H, H2), 2.27 (dd, *J* = 8.6, 6.4 Hz, 1H, H2), 2.92-2.96 (m, 1H, H1), 3.76 (s, 3H, H11), 3.82-3.94 (m, 1H, H5), 6.75-6.82 (m, 2H, H-Ar), 7.11-7.19 (m, 2H, H-Ar).

^{13}C-NMR (150 MHz; $CDCl_3$): δ 13.8 (C6), 21.9 (C2), 36.3 (C)1, 44.2 (C3), 55.2 (C11), 61.4 (C5), 113.5 (C-Ar), 126.4 (C-Ar), 130.0 (C-Ar), 158.8 (C-Ar), 167.4 (C4).

GC-MS (R_t, *m/z*): 24 min, 254 (M^+).

MS (EI) *m/z* (relative intensity): 254 (M^+, 57%), 225 (61), 189 (65), 145 (100).

HR-MS: 254.0711; calc for $C_{13}H_{15}O_3Cl$: 254.0710 (-0.6 ppm).

This compound has previously been reported in the literature.[1]

Ethyl 1-bromo-2-(4-(trifluoromethyl)phenyl)cyclopropane-1-carboxylate

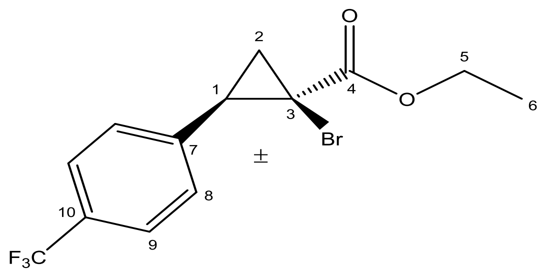

Catalytic: **2b** (0.597 mmol, 1.0 equiv), 4-trifluormethylstyrene (206 mg, 1.20 mmol, 2.0 equiv) and $Rh_2(esp)_2$ (6.4 mg, 0.0084 mmol, 0.01 equiv) gave 90.4 mg (0.268 mmol, 45%, dr 14:1) of ethyl 1-bromo-2-(4-(trifluoromethyl)phenyl)cyclopropane-1-carboxylate as a colorless oil.

Thermal: **2b** (0.597 mmol, 1.0 equiv) and 4-trifluormethylstyrene (206 mg, 1.20 mmol, 2.0 equiv) gave 69.2 mg (0.205 mmol, 34%, dr 3:1) of ethyl 1-bromo-2-(4-(trifluoromethyl)phenyl)cyclo-propane-1-carboxylate as a colorless oil.

Major diastereomer (structure displayed in drawing):

^{1}H-NMR (600 MHz; $CDCl_3$): δ 1.32 (t, *J* = 7.1 Hz, 3H, H6). 1.79 (dd, *J* = 8.4, 6.2 Hz, 1H, H2), 2.23 (dd, *J* = 10.1, 6.2 Hz, 1H, H2), 2.97 (dd, *J* = 10.1, 8.4 Hz, 1H, H1), 4.26 (dq, *J* = 7.1, 1.6 Hz, 2H, H5), 7.36-7.30 (m, 2H, H-Ar), 7.62-7.55 (m, 2H, H-Ar).

^{13}C-NMR (150 MHz; $CDCl_3$): δ 14.2 (C6), 24.0 (C2), 32.6 (C1), 34.7 (C3), 63.0 (C5), 125.1 (q, J=3.8 Hz, C-Ar), 129.8 (C-Ar), 129.8 (C-Ar), 139.7 (C-Ar), 169.5 (C4).

GC-MS (R_t, *m/z*): 22 min, 336/338 ($[M/M+2]^+$).

Minor diastereomer (structure not displayed):

^{1}H-NMR (600 MHz; $CDCl_3$): δ 0.87 (t, *J* = 7.1 Hz, 3H, H6), 1.83 (dd, *J* = 9.9, 6.8 Hz, 1H, H2), 2.37 (dd, *J* = 8.4, 6.8 Hz, 1H, H2), 3.10-3.04 (m, 1H, H1), 3.83-3.90 (m, 2H, H5), 7.36-7.30 (m, 2H, H-Ar), 7.52 (d, *J* = 8.1 Hz, 2H, H-Ar).

^{13}C-NMR (150 MHz; $CDCl_3$): δ 13.6 (C6), 22.2 (C2), 31.4 (C1), 36.2 (C3), 62.4 (C5), 125.0-125.1 (m, C11), 129.2 (C-Ar), 129.4 (C-Ar), 138.6 (C-Ar), 166.6 (C4).

GC-MS (R_t, *m/z*): 21 min, 336/338 ($[M/M+2]^+$).

MS (EI) *m/z* (relative intensity): 336/338 ($[M/M+2]^+$, 61%/61), 227/229 (15/20), 183 (100), 115 (72).

HR-MS: 335.9969; calc for $C_{13}H_{12}O_2F_3{}^{79}Br$: 335.9973 (1.1 ppm).

This compound has previously been reported in the literature.[1]

Ethyl 1-chloro-2-(4-(trifluormethyl)phenyl)cyclopropane-1-carboxylate

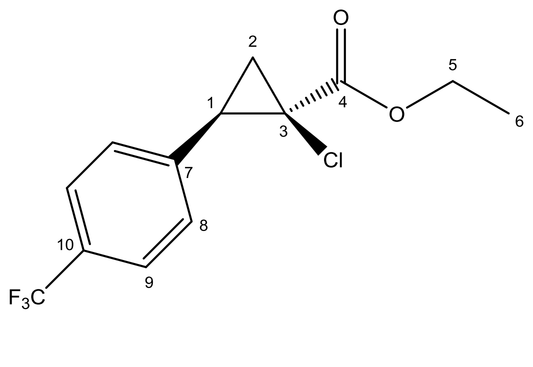

Catalytic: **2a** (0.597 mmol, 1.0 equiv), 4-trifluoromethylstyrene (206 mg, 1.20 mmol, 2.0 equiv) and $Rh_2(esp)_2$ (7.6 mg, 0.010 mmol, 0.02 equiv) gave 75.1 mg (0.257 mmol, 43%, dr 24:1) of ethyl 1-chloro-2-(4-(trifluormethyl)phenyl)cyclopropane-1-carboxylate as a colorless oil.

Thermal: **2a** (0.597 mmol, 1.0 equiv) and 4-trifluoromethylstyrene (206 mg, 1.20 mmol) gave 54.4 mg (0.186 mmol, 31%, dr 5:1) of ethyl 1-chloro-2-(4-(trifluormethyl)phenyl)cyclopropane-1-carboxylate as a colorless oil.

Major diastereomer (structure displayed in drawing):

^{1}H-NMR (600 MHz; $CDCl_3$): δ 1.33 (t, *J* = 7.1 Hz, 3H, H6). 1.76 (dd, *J* = 8.5, 6.1 Hz, 1H, H2), 2.18 (dd, *J* = 10.1, 6.1 Hz, 1H, H2), 3.09 (dd, *J* = 10.1, 8.5 Hz, 1H, H1), 4.27 (dq, *J* = 7.1, 1.4 Hz, 2H, H5), 7.34 (d, *J* = 8.1 Hz, 2H, H-Ar), 7.58 (d, *J* = 8.1 Hz, 2H, H-Ar).

^{13}C-NMR (150 MHz; $CDCl_3$): 14.1 (C6), 23.6 (C2), 33.1 (C1), 44.6 (C3), 62.9 (C5), 125.10 (q, *J* = 3.7 Hz, C-Ar), 126.8 (C-Ar), 129.8 (C-Ar), 138.6 (C-Ar), 169.8 (C4).

GC-MS (R_t, *m/z*): 22 min, 292 (M^+).

Due to the high diasteromeric ratio, the minor isomer was not characterized.

MS (EI) *m/z* (relative intensity): 292/294 (M^+, 76%), 264 (65), 183 (100), 115 (51).

HR-MS: 292.0469; calc for $C_{13}H_{12}O_2F_3Cl$: 292.0478 (3.2 ppm)

This compound has not previously been reported in the literature.

^{1}H NMR and ^{13}C NMR spectra

Ethyl bromodiazoacetate (2b)

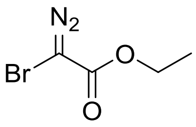

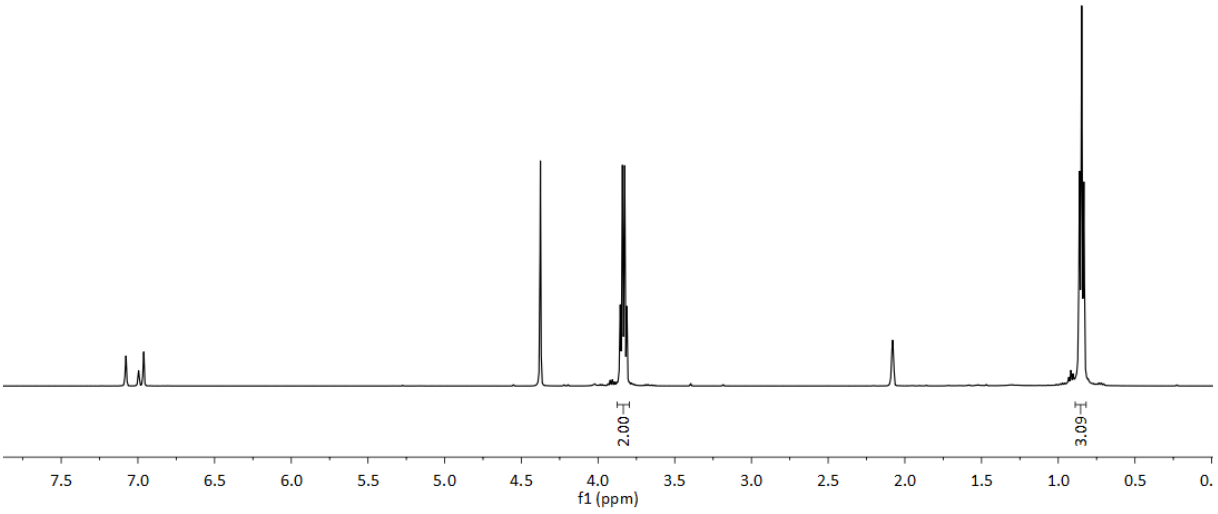

^{1}H NMR-spectrum (500 MHz, toluene-d_8)

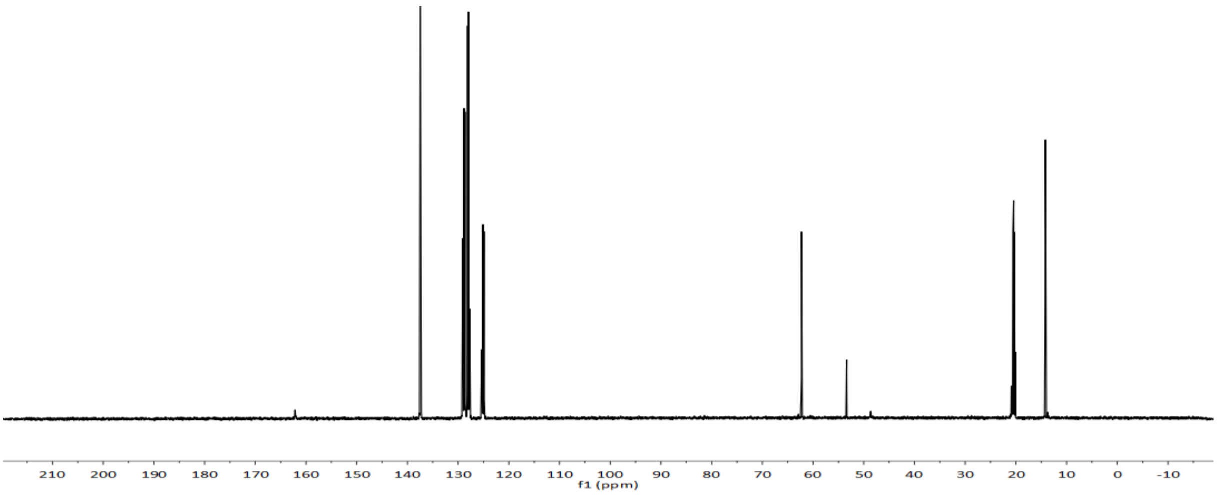

^{13}C NMR-spectrum (125 MHz, toluene-d_8)

Ethyl chlorodiazoacetate (2a)

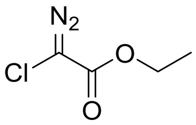

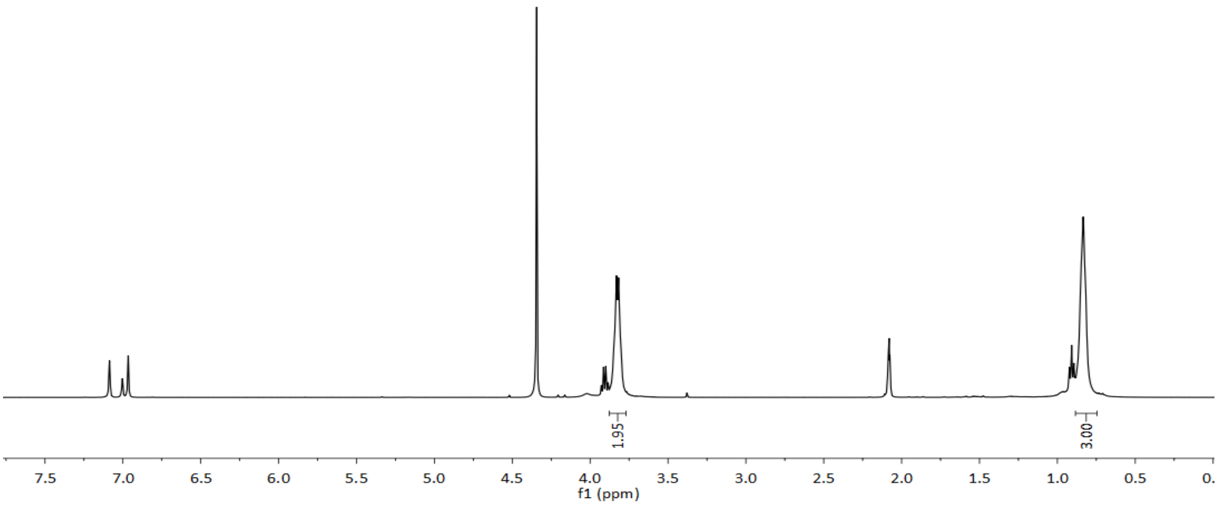

^{1}H NMR spectrum (500 MHz, toluene-d_8).

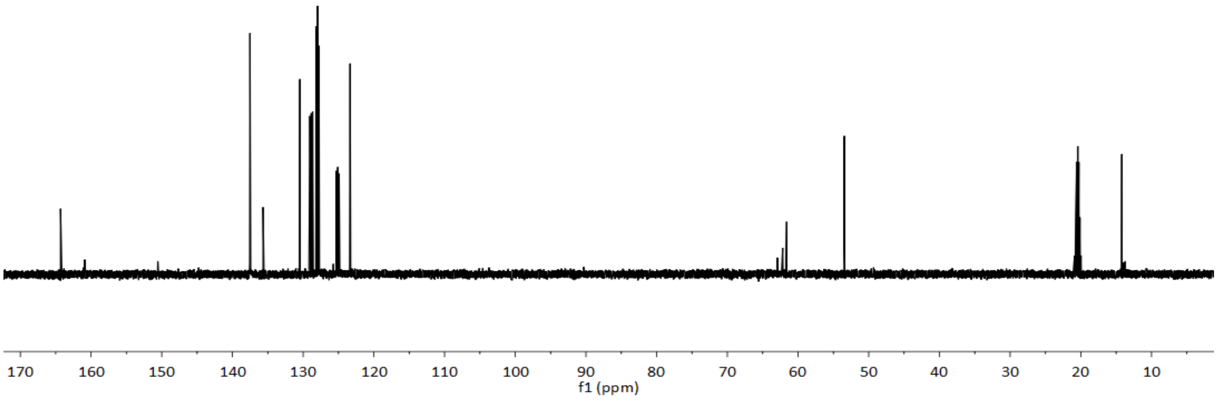

^{13}C NMR spectrum (125 MHz, toluene-d_8) containing internal standard (ethyl 4-nitrobenzoate).

Ethyl iododiazoacetate (2c)

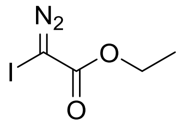

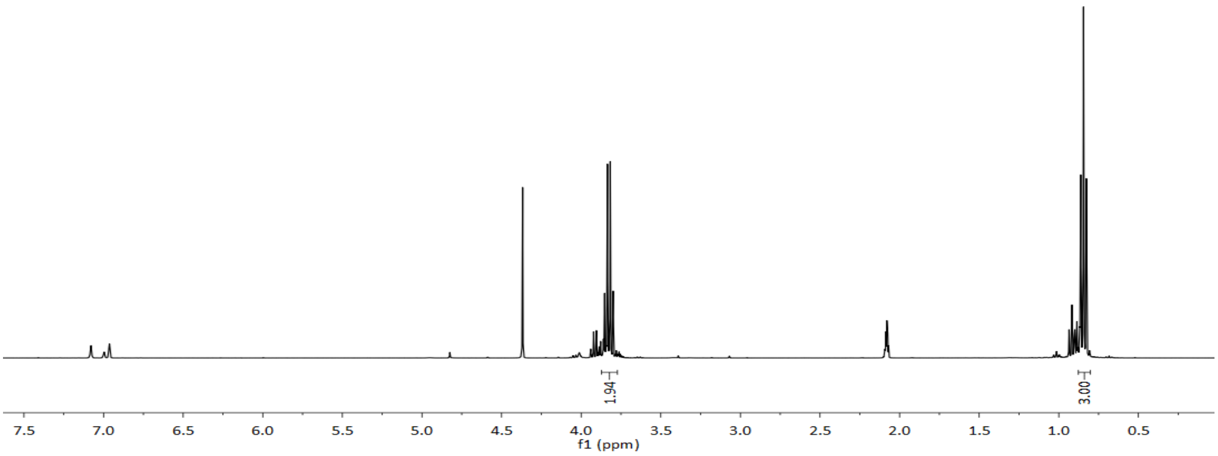

^{1}H NMR-spectrum (400 MHz, toluene-d_8).

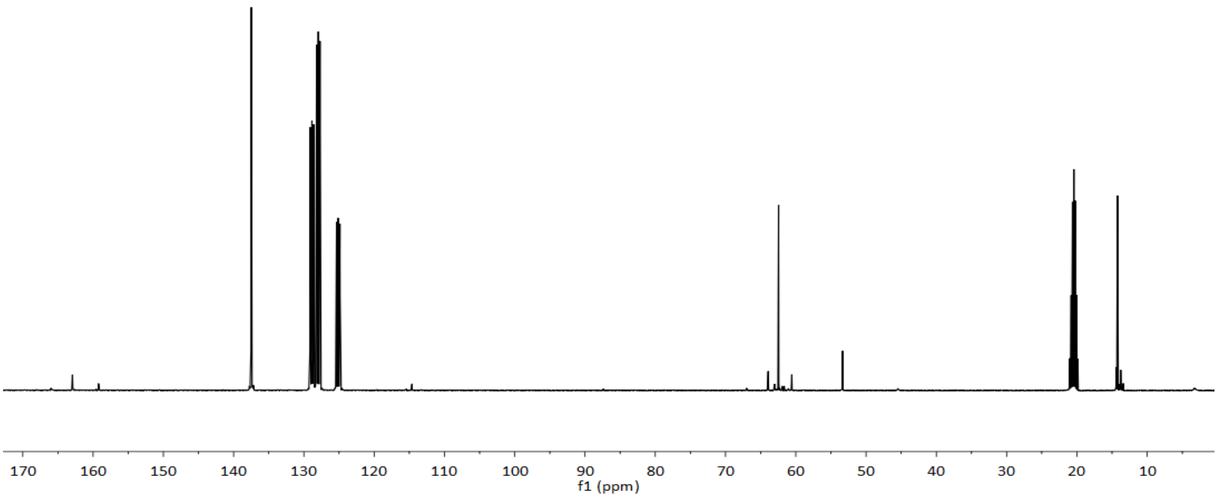

^{13}C NMR spectrum (100 MHz, toluene-d_8).

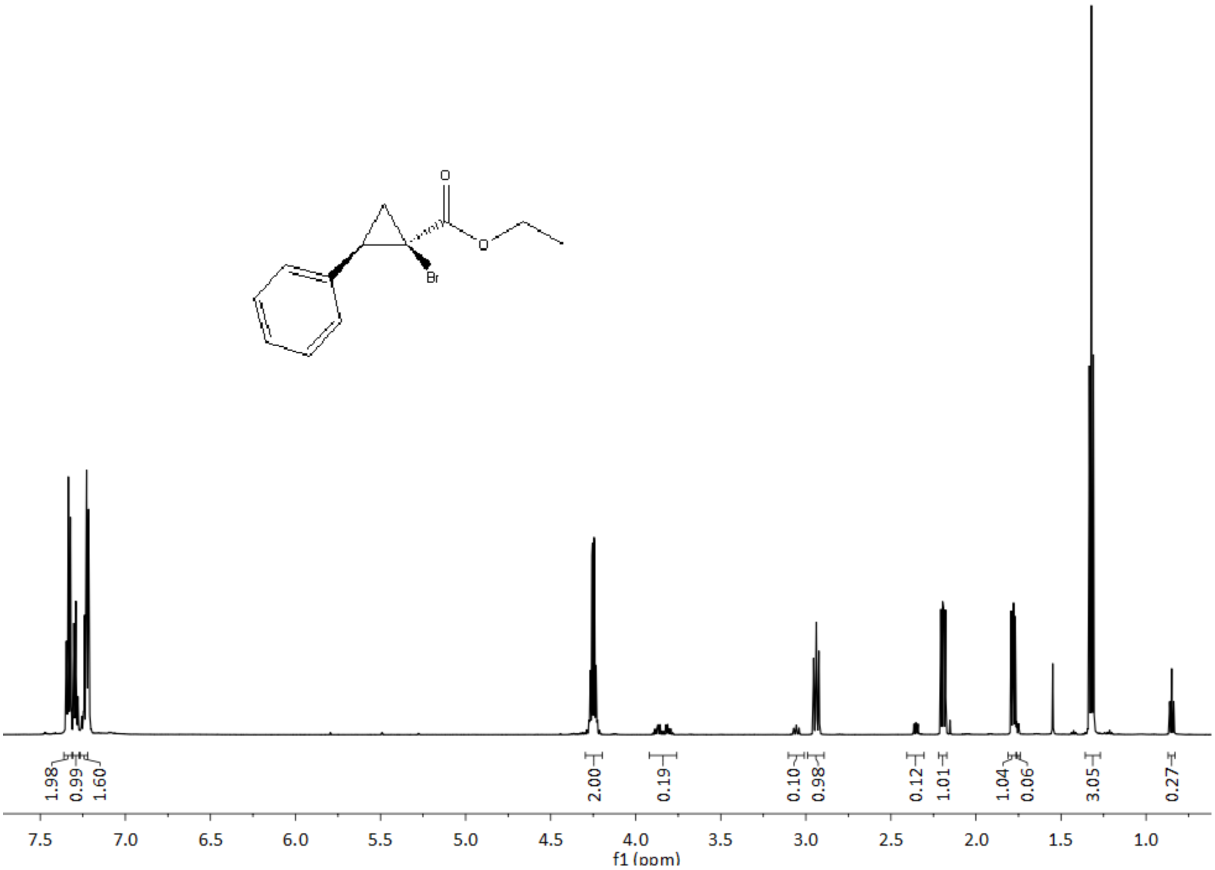

^{1}H NMR spectrum (600 MHz, $CDCl_3$), mixture of diastereomers.

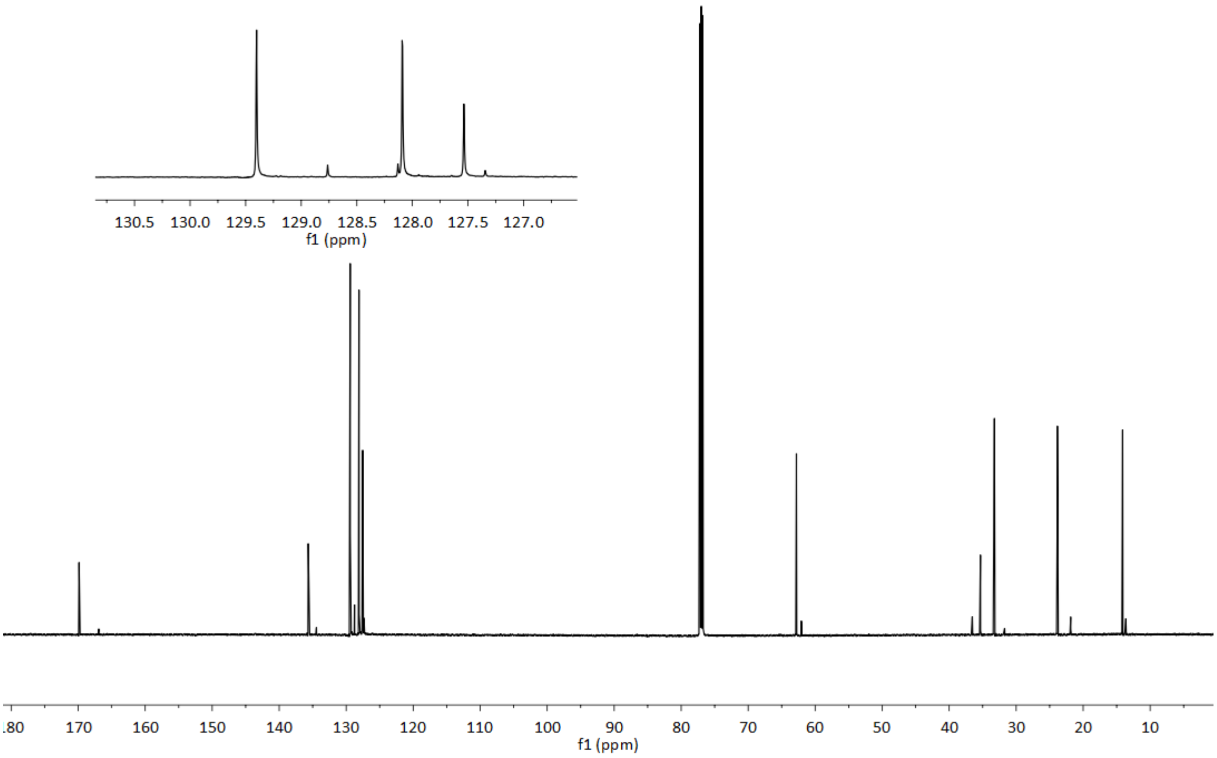

^{13}C NMR spectrum (150 MHz, $CDCl_3$), mixture of diastereomers.

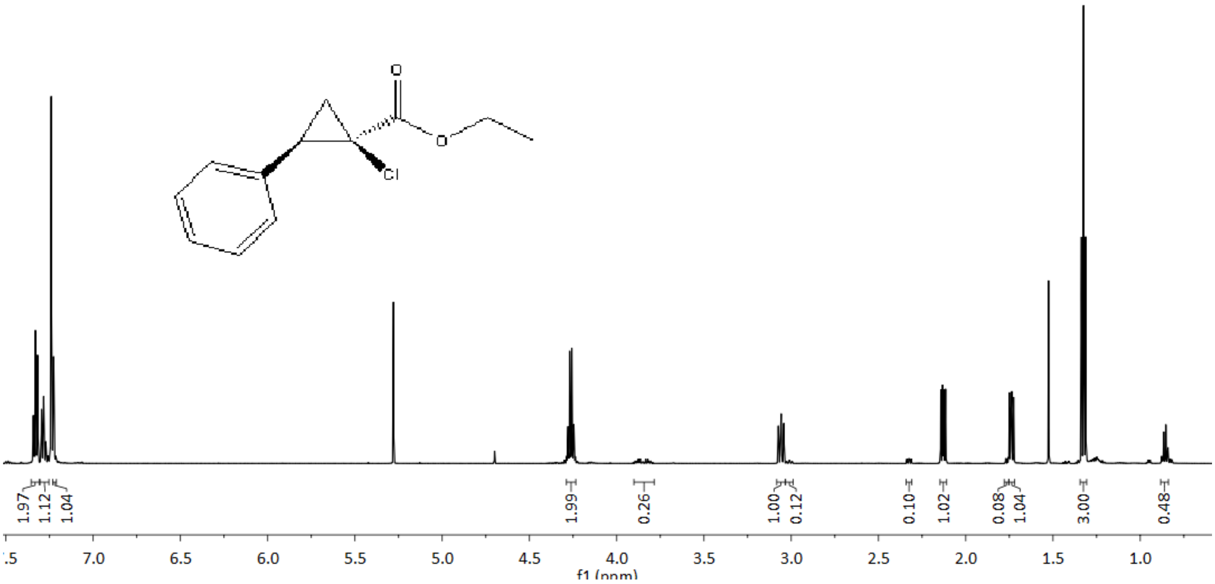

^{1}H NMR spectrum (600 MHz, $CDCl_3$), mixture of diastereomers.

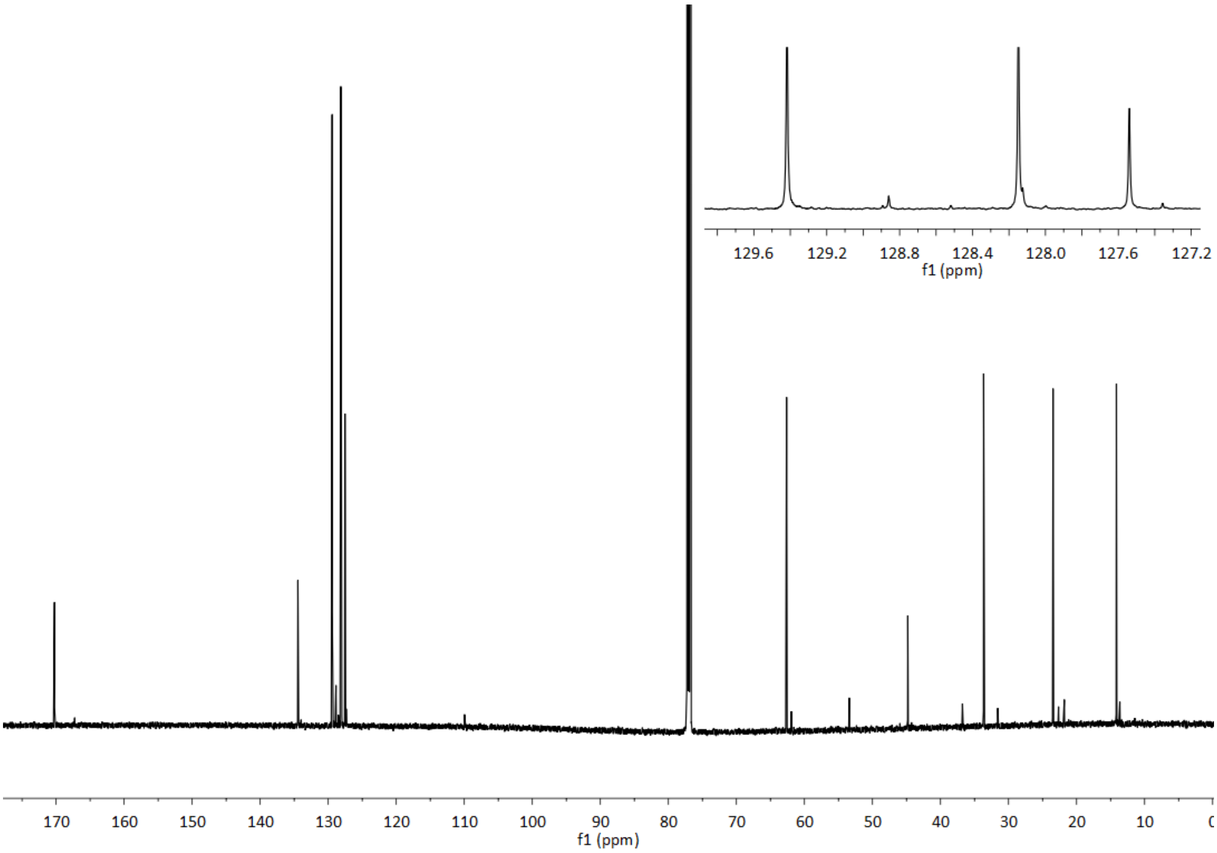

^{13}C NMR- spectrum (150 MHz, $CDCl_3$), mixture of diastereomers.

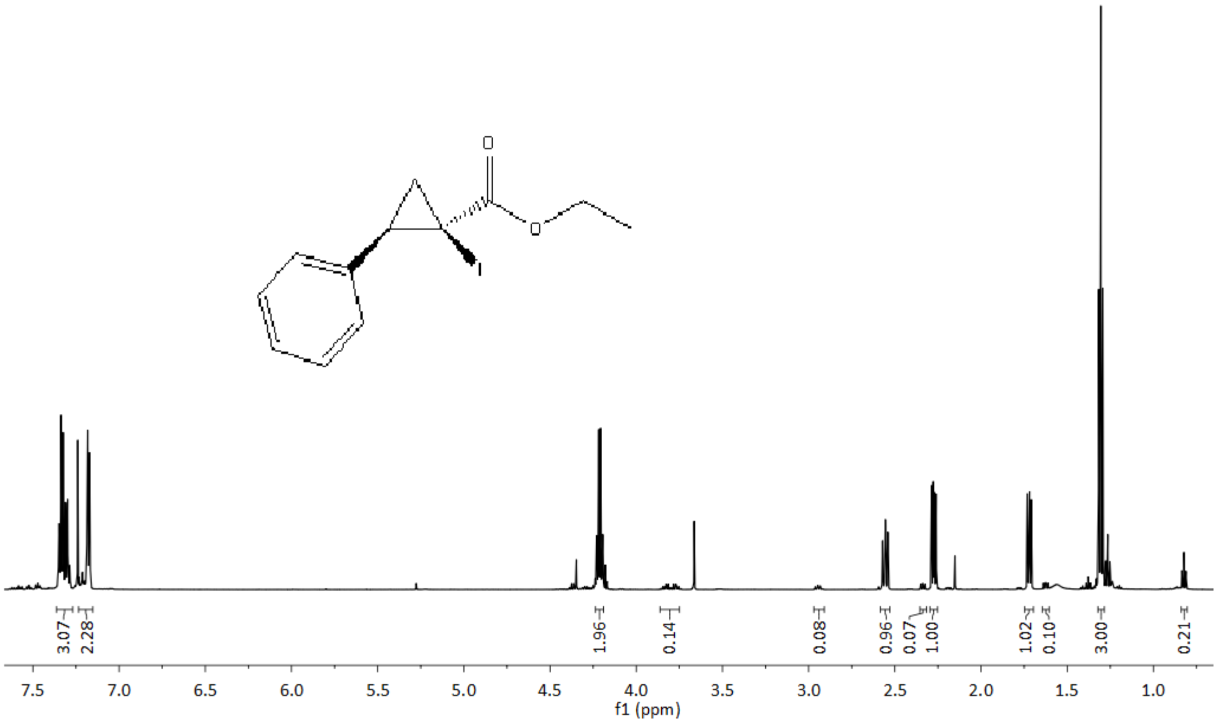

^{1}H NMR-spectrum (600 MHz, $CDCl_3$), mixture of diastereomers.

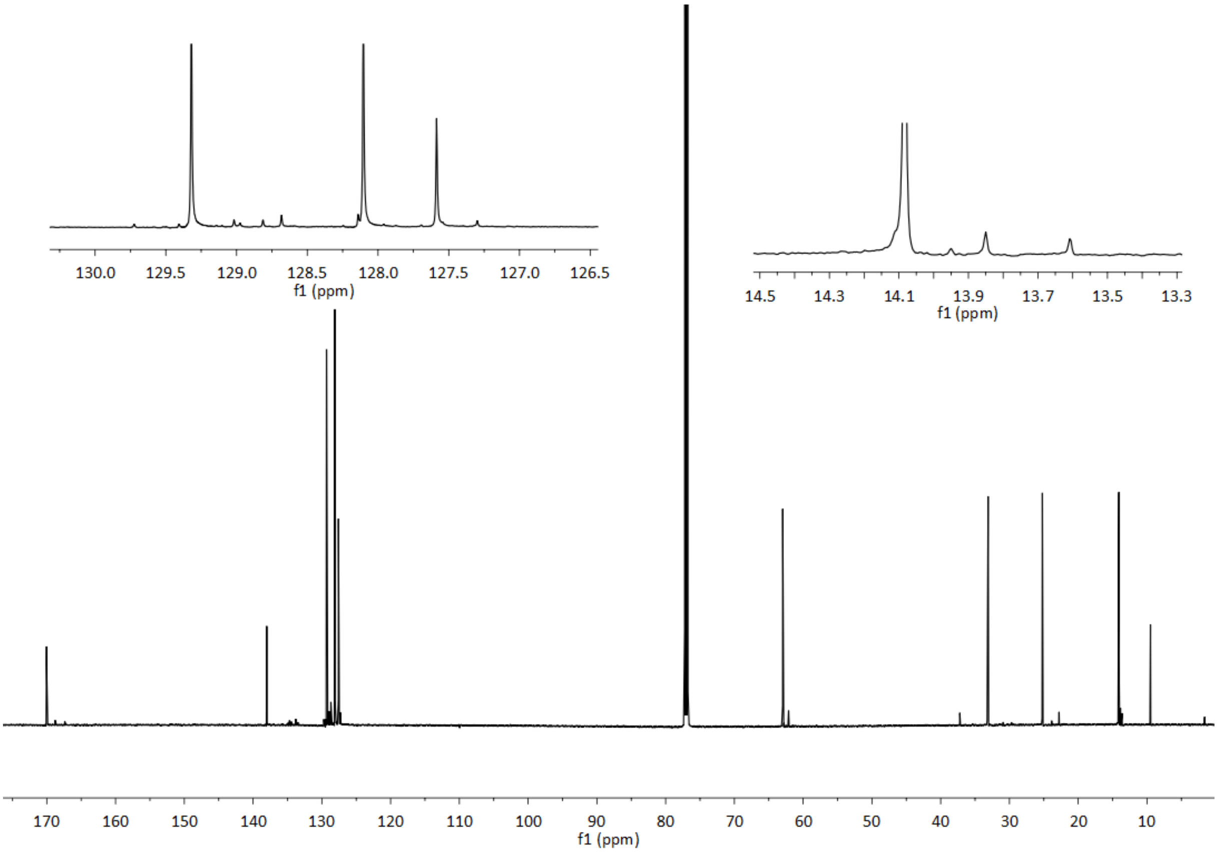

^{13}C NMR spectrum (150 MHz, $CDCl_3$), mixture of diastereomers.

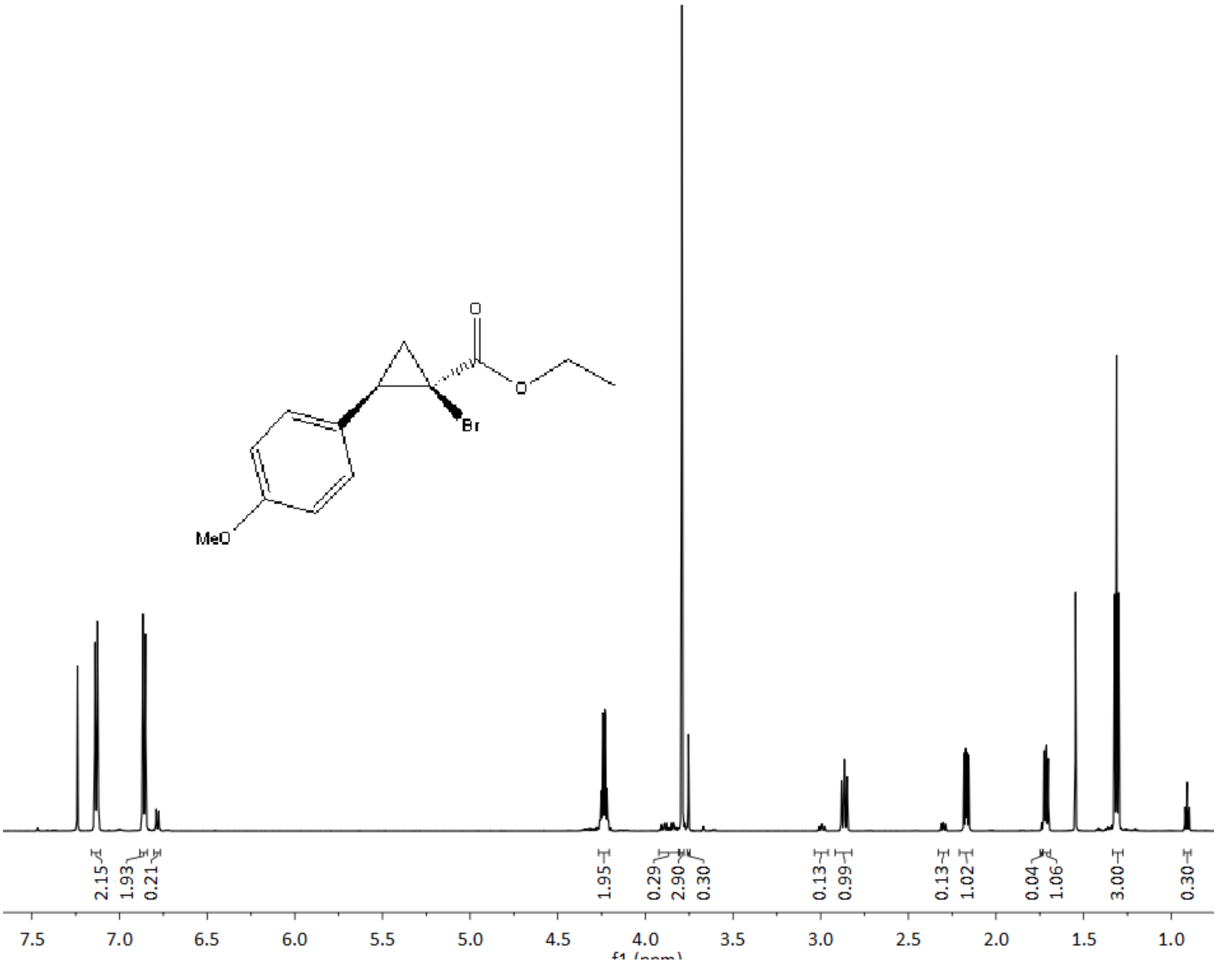

^{1}H NMR-spectrum (600 MHz, $CDCl_3$), mixture of diastereomers.

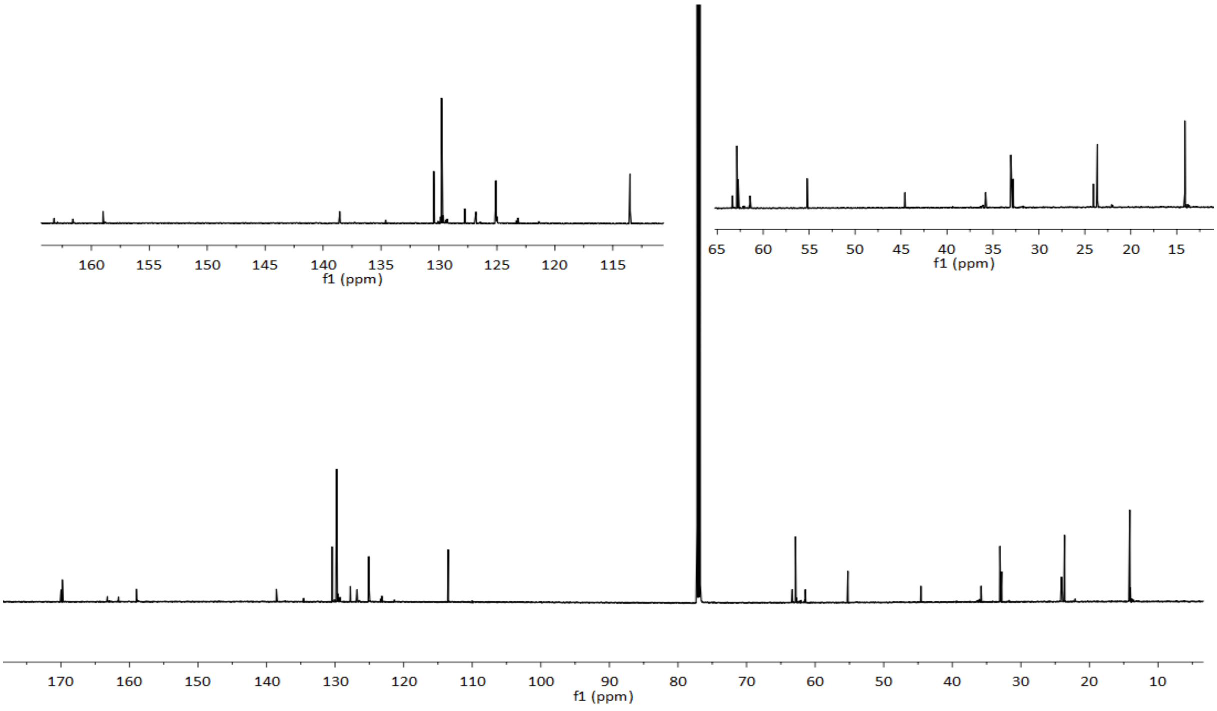

^{13}C NMR spectrum (150 MHz, $CDCl_3$), mixture of diastereomers.

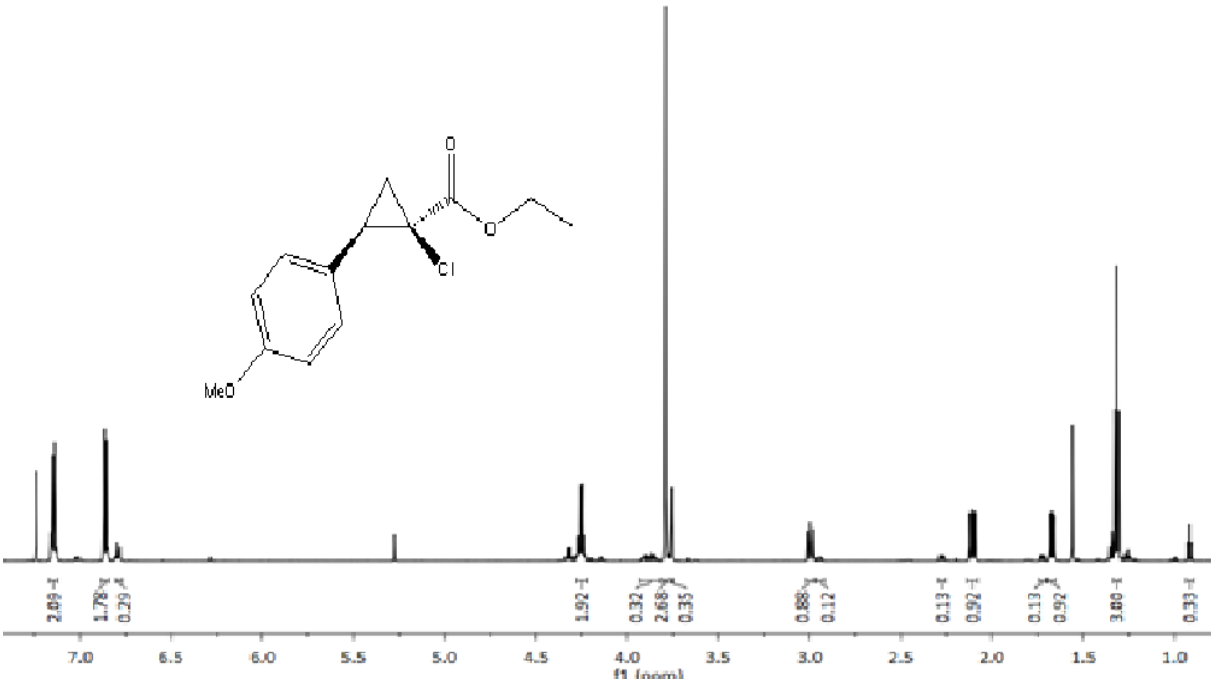

^{1}H NMR spectrum (600 MHz, $CDCl_3$), mixture of diastereomers.

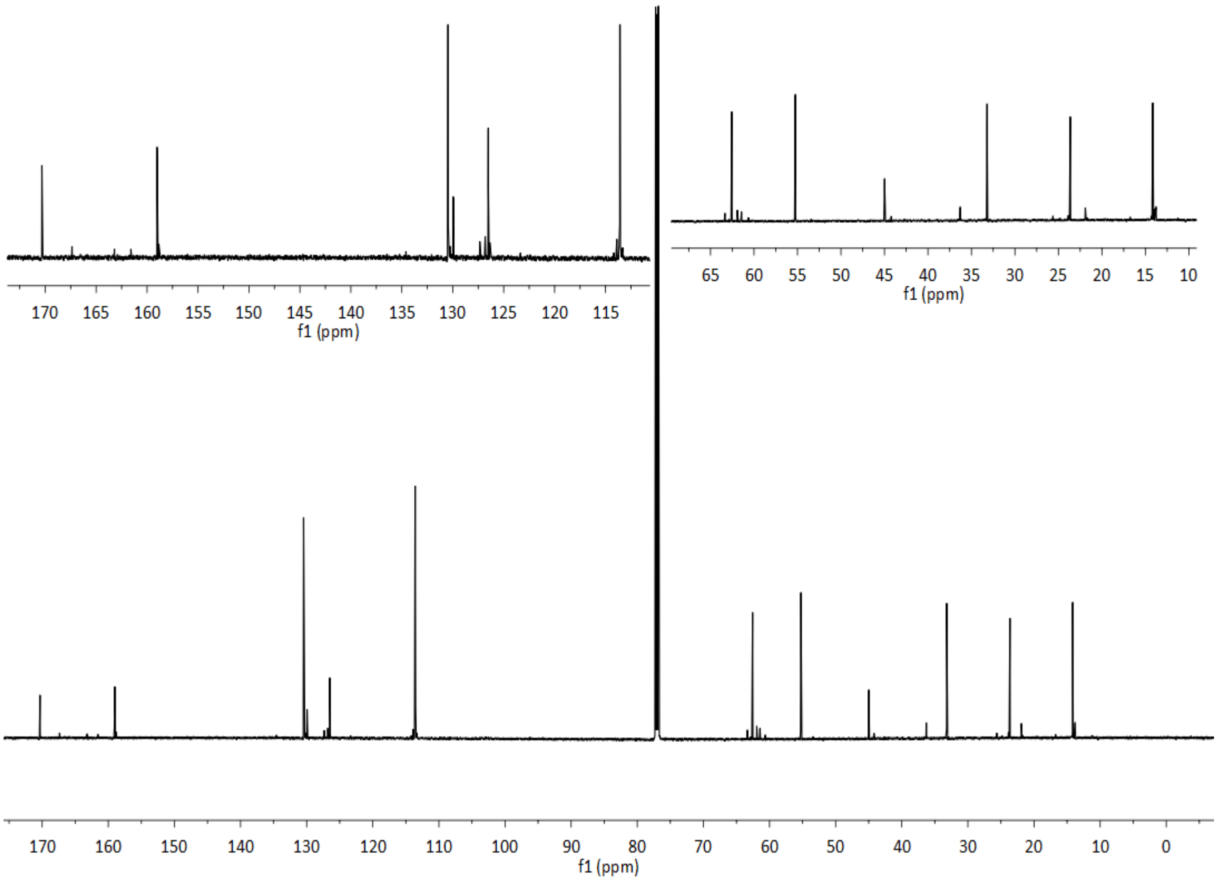

^{1}H NMR spectrum (600 MHz, $CDCl_3$), mixture of diastereomers.

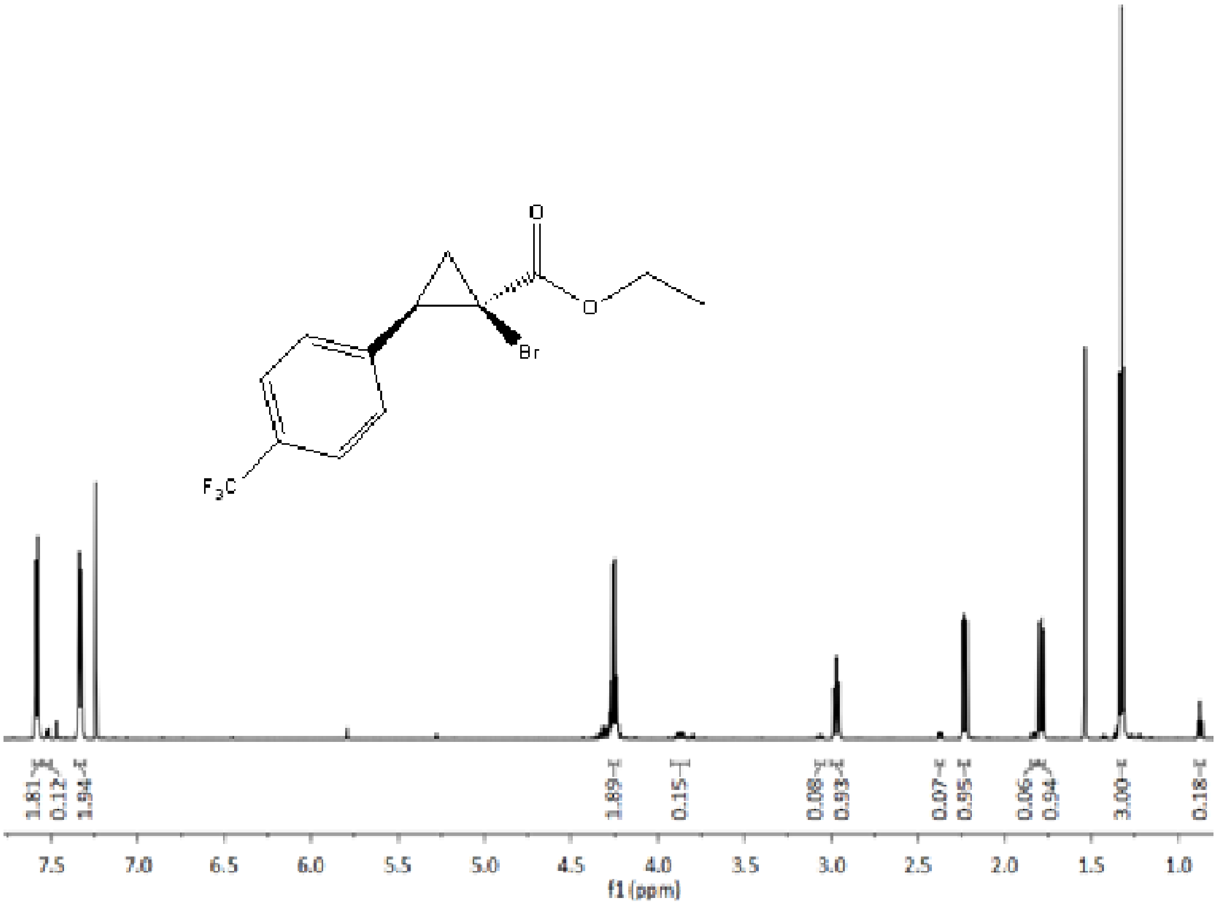

^{1}H NMR spectrum (600 MHz, $CDCl_3$), mixture of diastereomers.

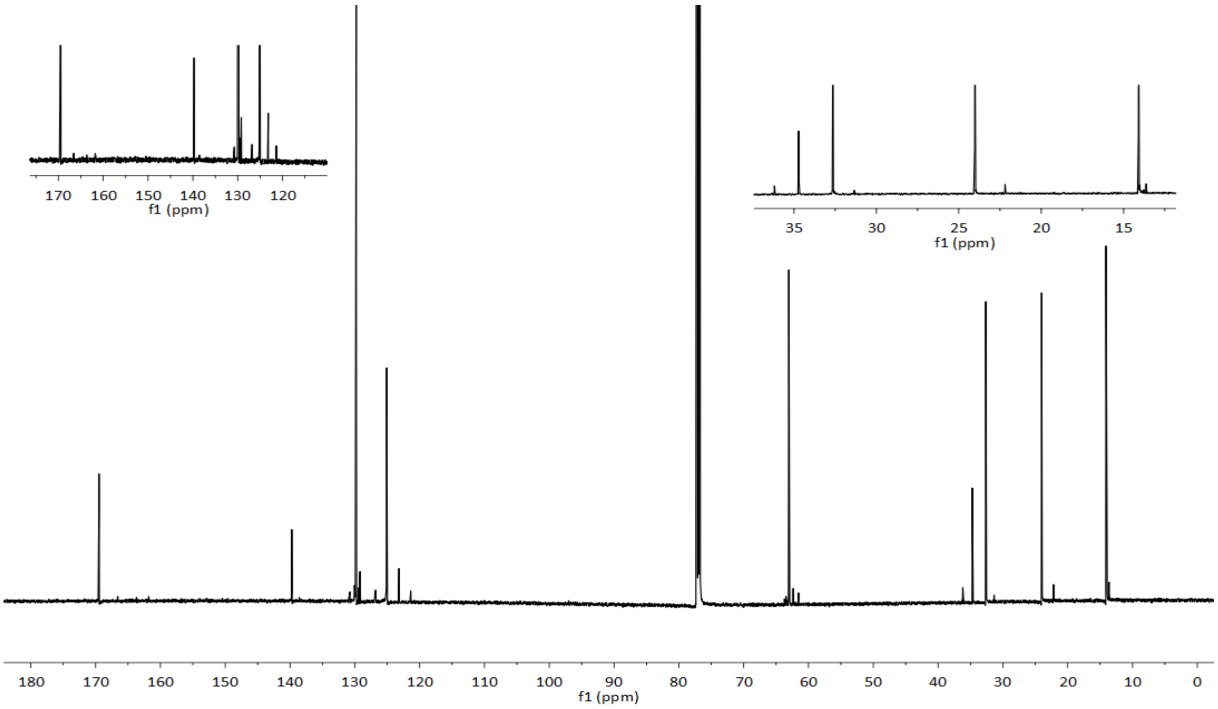

^{13}C NMR spectrum (150 MHz, $CDCl_3$), mixture of diastereomers.

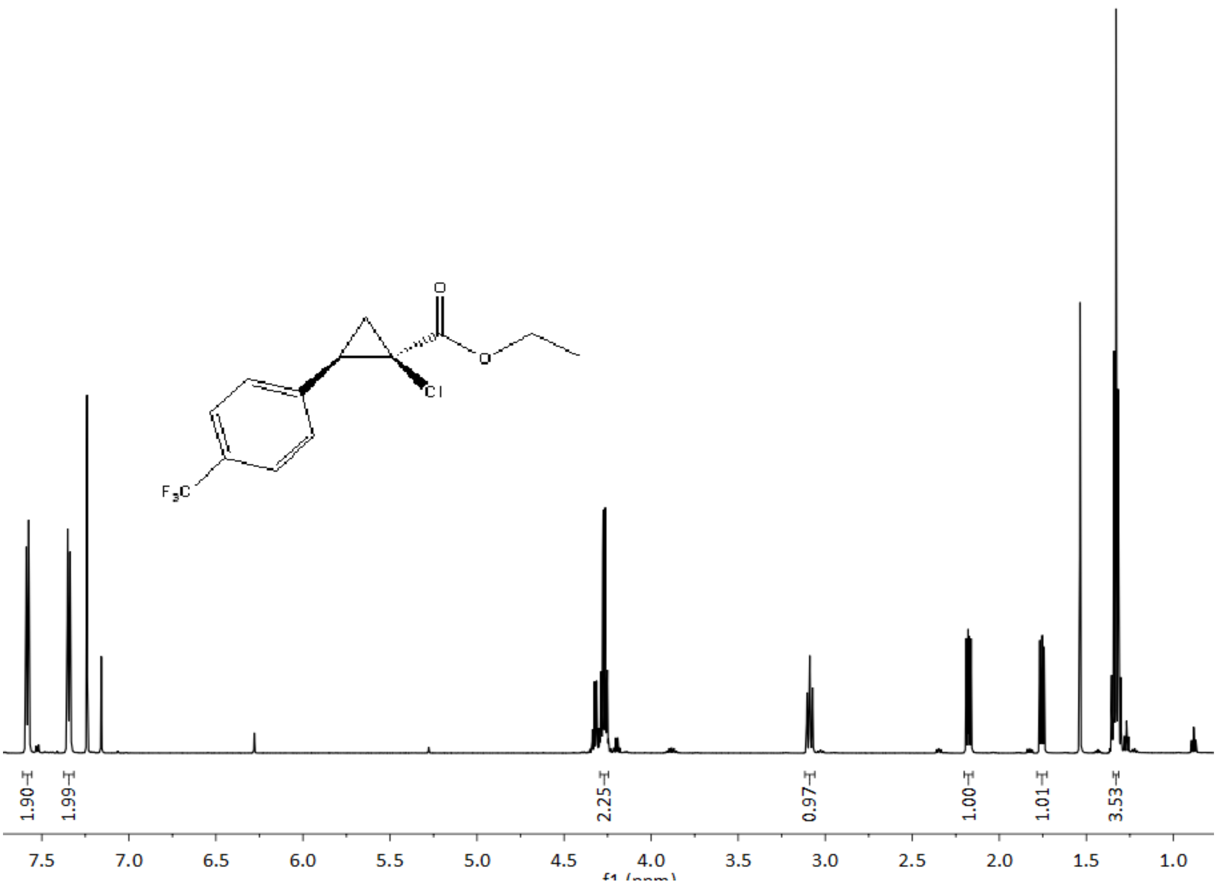

^{1}H NMR spectrum (600 MHz, $CDCl_3$), mixture of diastereomers.

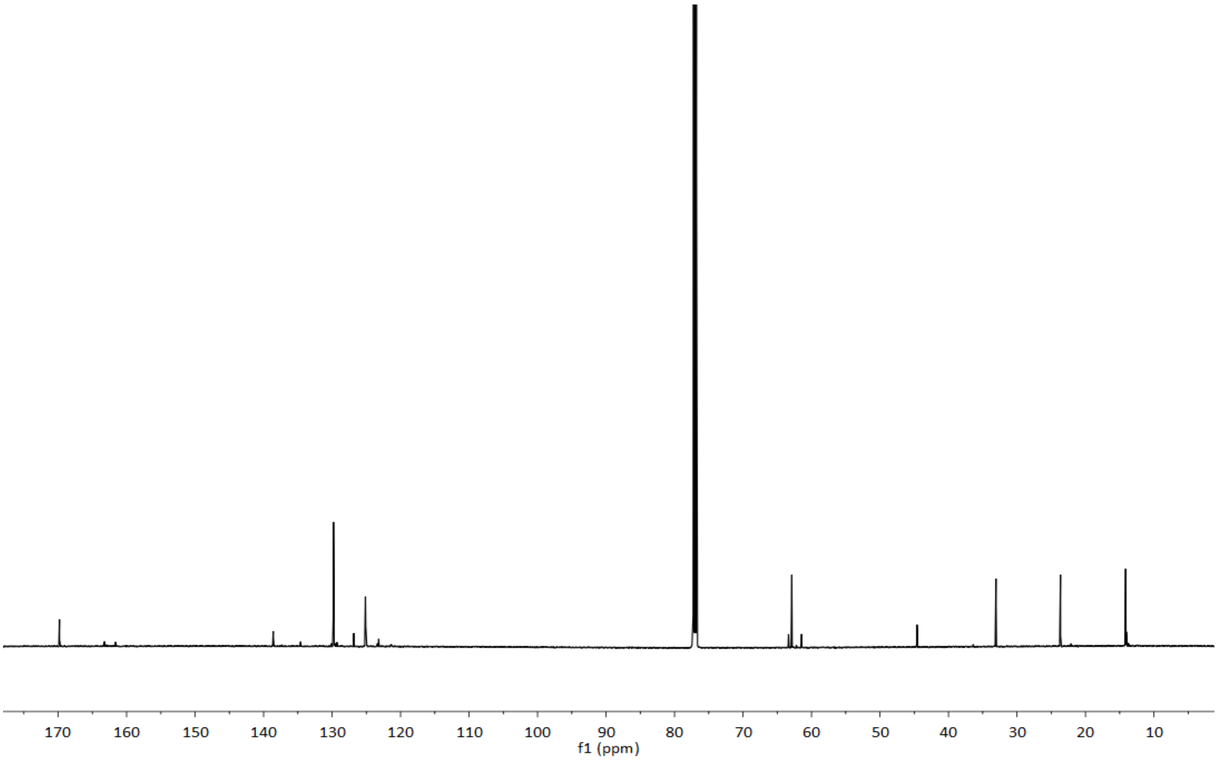

^{13}C NMR spectrum (150 MHz, $CDCl_3$), mixture of diastereomers.

References

1. Bonge, H. T.; Pintea, B.; Hansen, T. *Org. Biomol. Chem.* **2008**, *6*, 3670-3672.
