# Supplementary material for: On the cause of low thermal stability of ethyl halodiazoacetates
Source: Beilstein J Org Chem. 2016 Jul 26;12:1590–7. doi: 10.3762/bjoc.12.155 (PMC4979875; doi:10.3762/bjoc.12.155)
Supplement: File 2 — DFT calculations. [file Beilstein_J_Org_Chem-12-1590-s002.pdf]

Supporting Information File 2

for

On the cause of low thermal stability of ethyl halodiazoacetates

Magnus Mortén, Martin Hennum and Tore Bonge-Hansen*

Address: Department of Chemistry, University of Oslo, P.O. Box 1033 Blindern, NO-0315 Oslo, Norway

Email: Tore Bonge-Hansen* - tore.hansen@kjemi.uio.no

*Corresponding author

DFT calculations

General information S2

Ethyl diazoacetate (**1**) S3

Diethyl diazomalonate (**2e**) S7

Ethyl diazofluoroacetate (**2d**) S11

Ethyl chlorodiazoacetate (**2a**) S15

Ethyl bromodiazoacetate (**2b**) S19

General information

All calculations were performed with the Gaussian 09 program, using the hybrid functional B3LYP. All geometry optimizations were carried out with a cc-pVTZ basis set for all atoms without any constraint. Solvation effects (toluene) were included during the optimization with the SMD method, as implemented in Gaussian 09. Each stationary point was identified as either a minimum or a saddle point by analytical calculation of the frequencies. The connectivity among reactant, transition state (TS), and product was verified by following the intrinsic reaction coordinates (IRC). The Gibbs energies were calculated within the harmonic approximation of frequencies at T = 298.15 K and p = 1 atm. The NBO analysis was carried out using NBO 3.1.

Ethyl diazoacetate (1)

Zero-point correction=	0.104291 (Hartree/Particle)
Thermal correction to Energy=	0.112809
Thermal correction to Enthalpy=	0.113753
Thermal correction to Gibbs Free Energy=	0.070602
Sum of electronic and zero-point Energies=	-416.009457
Sum of electronic and thermal Energies=	-416.000940
Sum of electronic and thermal Enthalpies=	-415.999995
Sum of electronic and thermal Free Energies=	-416.043146

Center Number	Atomic Number	Atomic Type	Coordinates (Angstroms) X	Y	Z
1	6	0	0.227051	0.187984	0.000000
2	8	0	0.384074	1.391884	0.000005
3	8	0	-0.970666	-0.427581	-0.000002
4	6	0	1.294599	-0.797199	-0.000004
5	6	0	-2.138553	0.432013	0.000004
6	1	0	-2.100935	1.070526	-0.882344
7	1	0	-2.100933	1.070518	0.882357
8	6	0	-3.361597	-0.452785	0.000001
9	1	0	-3.390334	-1.088395	0.885498
10	1	0	-4.257365	0.170290	0.000008
11	1	0	-3.390339	-1.088384	-0.885504
12	7	0	2.521368	-0.356475	-0.000003
13	7	0	3.571205	0.041604	-0.000002
14	1	0	1.155631	-1.864958	-0.000009

Ethyl diazoacetate TS

Zero-point correction=	0.099313 (Hartree/Particle)
Thermal correction to Energy=	0.108843
Thermal correction to Enthalpy=	0.109787
Thermal correction to Gibbs Free Energy=	0.063556
Sum of electronic and zero-point Energies=	-415.948224
Sum of electronic and thermal Energies=	-415.938694
Sum of electronic and thermal Enthalpies=	-415.937750
Sum of electronic and thermal Free Energies=	-415.983981

Center Number	Atomic Number	Atomic Type	Coordinates (Angstroms) X	Y	Z
1	6	0	-0.044204	1.080516	-0.004732
2	8	0	-0.668235	2.144373	-0.011579
3	8	0	-0.546349	-0.137069	0.013949
4	6	0	1.323149	1.412950	-0.177832
5	6	0	-2.006078	-0.244430	-0.000236
6	1	0	-2.373813	0.259198	-0.892925
7	1	0	-2.395246	0.271571	0.876178
8	6	0	-2.354116	-1.711667	0.006739
9	1	0	-1.971059	-2.204513	0.900412
10	1	0	-3.439314	-1.821592	-0.004104
11	1	0	-1.951626	-2.217083	-0.871130
12	7	0	2.461447	-0.654125	0.156388
13	7	0	3.045809	-1.533628	-0.117306
14	1	0	1.784437	1.744042	0.755406

Singlet carbene from ethyl diazoacetate

Zero-point correction=	0.092229 (Hartree/Particle)
Thermal correction to Energy=	0.099126
Thermal correction to Enthalpy=	0.100070
Thermal correction to Gibbs Free Energy=	0.061374
Sum of electronic and zero-point Energies=	-306.388564
Sum of electronic and thermal Energies=	-306.381667
Sum of electronic and thermal Enthalpies=	-306.380723
Sum of electronic and thermal Free Energies=	-306.419419

Center Number	Atomic Number	Atomic Type	Coordinates (Angstroms) X	Y	Z
1	6	0	-1.072939	-0.104571	0.001920
2	8	0	-1.480189	1.068066	-0.022936
3	8	0	0.158907	-0.538078	0.046623
4	6	0	-2.269807	-0.812312	-0.168391
5	6	0	1.223668	0.473867	0.045390
6	1	0	1.134627	1.056258	0.961147
7	1	0	1.063407	1.130674	-0.807662
8	6	0	2.540156	-0.254934	-0.037245
9	1	0	2.610293	-0.840287	-0.953894
10	1	0	3.350505	0.475313	-0.035641
11	1	0	2.679186	-0.919885	0.815085
12	1	0	-2.794220	-0.954281	0.781422

Triplet carbene from ethyl diazoacetate

Zero-point correction=	0.092086 (Hartree/Particle)
Thermal correction to Energy=	0.098893
Thermal correction to Enthalpy=	0.099837
Thermal correction to Gibbs Free Energy=	0.060107
Sum of electronic and zero-point Energies=	-306.394040
Sum of electronic and thermal Energies=	-306.387232
Sum of electronic and thermal Enthalpies=	-306.386288
Sum of electronic and thermal Free Energies=	-306.426018

Center Number	Atomic Number	Atomic Type	Coordinates (Angstroms) X	Y	Z
1	6	0	1.122983	-0.012579	-0.000001
2	8	0	1.399688	1.178779	-0.000002
3	8	0	-0.122699	-0.504189	0.000000
4	6	0	2.142454	-1.014193	0.000000
5	6	0	-1.207307	0.462293	0.000000
6	1	0	-1.107519	1.093987	-0.882745
7	1	0	-1.107515	1.093991	0.882741
8	6	0	-2.506694	-0.304665	0.000003
9	1	0	-2.593179	-0.934566	0.885561
10	1	0	-3.339925	0.399426	0.000003
11	1	0	-2.593183	-0.934570	-0.885552
12	1	0	3.216784	-0.900134	0.000000

Diethyl diazomalonate (2e)

Zero-point correction=	0.175817 (Hartree/Particle)
Thermal correction to Energy=	0.189913
Thermal correction to Enthalpy=	0.190857
Thermal correction to Gibbs Free Energy=	0.133860
Sum of electronic and zero-point Energies=	-683.238948
Sum of electronic and thermal Energies=	-683.224852
Sum of electronic and thermal Enthalpies=	-683.223907
Sum of electronic and thermal Free Energies=	-683.280905

Center Number	Atomic Number	Atomic Type	Coordinates (Angstroms) X	Y	Z
1	6	0	-0.000001	-0.022558	0.000000
2	6	0	-1.308499	-0.696582	0.039258
3	6	0	1.308497	-0.696584	-0.039258
4	7	0	0.000001	1.295478	0.000000
5	7	0	0.000012	2.413047	0.000000
6	8	0	-1.465540	-1.889731	0.088660
7	8	0	1.465537	-1.889733	-0.088660
8	8	0	2.307935	0.208855	-0.014987
9	8	0	-2.307937	0.208857	0.014987
10	6	0	3.661409	-0.315883	-0.065563
11	1	0	3.796038	-0.829705	-1.017496
12	1	0	3.779566	-1.044954	0.734737
13	6	0	-3.661411	-0.315881	0.065564
14	1	0	-3.796040	-0.829703	1.017497
15	1	0	-3.779568	-1.044952	-0.734737
16	6	0	-4.611396	0.846500	-0.087282
17	1	0	-4.464160	1.354315	-1.040909
18	1	0	-5.637880	0.478580	-0.053470
19	1	0	-4.484973	1.572411	0.716387
20	6	0	4.611394	0.846498	0.087282
21	1	0	4.464158	1.354314	1.040908
22	1	0	5.637878	0.478578	0.053470
23	1	0	4.484970	1.572409	-0.716388

Diethyl diazomalonate TS

Zero-point correction=	0.170802 (Hartree/Particle)
Thermal correction to Energy=	0.186089
Thermal correction to Enthalpy=	0.187033
Thermal correction to Gibbs Free Energy=	0.126227
Sum of electronic and zero-point Energies=	-683.183528
Sum of electronic and thermal Energies=	-683.168241
Sum of electronic and thermal Enthalpies=	-683.167297
Sum of electronic and thermal Free Energies=	-683.228104

Center Number	Atomic Number	Atomic Type	Coordinates (Angstroms) X	Y	Z
1	6	0	1.269021	-0.859822	-0.292887
2	8	0	1.541032	-2.046795	-0.287388
3	8	0	2.073499	0.127211	0.099104
4	6	0	0.001423	-0.499868	-0.873959
5	6	0	3.411156	-0.266454	0.529477
6	1	0	3.894681	-0.791667	-0.293175
7	1	0	3.315116	-0.953995	1.368562
8	6	0	4.152605	0.990396	0.912534
9	1	0	3.655711	1.509167	1.732552
10	1	0	5.159987	0.726609	1.237580
11	1	0	4.236429	1.673145	0.066842
12	7	0	0.004615	1.689080	-0.755046
13	7	0	0.007467	2.702388	-1.159767
14	6	0	-1.276732	-0.852469	-0.314277
15	8	0	-1.561529	-2.036290	-0.344792
16	8	0	-2.070704	0.132204	0.102633
17	6	0	-3.411574	-0.258816	0.526175
18	1	0	-3.319899	-0.965304	1.349937
19	1	0	-3.901111	-0.761953	-0.306599
20	6	0	-4.141488	0.995467	0.938583
21	1	0	-4.221991	1.697432	0.108467
22	1	0	-5.150064	0.732794	1.260796
23	1	0	-3.638292	1.492251	1.768394

Singlet carbene from diethyl diazomalonate

Zero-point correction=	0.163300 (Hartree/Particle)
Thermal correction to Energy=	0.175451
Thermal correction to Enthalpy=	0.176395
Thermal correction to Gibbs Free Energy=	0.123198
Sum of electronic and zero-point Energies=	-573.630016
Sum of electronic and thermal Energies=	-573.617865
Sum of electronic and thermal Enthalpies=	-573.616921
Sum of electronic and thermal Free Energies=	-573.670119

Center Number	Atomic Number	Atomic Type	Coordinates (Angstroms) X	Y	Z
1	6	0	-0.000001	-0.882505	0.885881
2	6	0	1.253445	-0.813207	0.198034
3	6	0	-1.253449	-0.813213	0.198035
4	8	0	1.650179	-1.849553	-0.305533
5	8	0	-1.650188	-1.849560	-0.305524
6	8	0	-1.880855	0.351309	0.251631
7	8	0	1.880858	0.351310	0.251638
8	6	0	-3.231845	0.410376	-0.312319
9	1	0	-3.834053	-0.356999	0.171493
10	1	0	-3.166702	0.184997	-1.375613
11	6	0	3.231843	0.410372	-0.312323
12	1	0	3.166689	0.185003	-1.375618
13	1	0	3.834050	-0.357013	0.171477
14	6	0	3.765100	1.797310	-0.057252
15	1	0	3.810057	2.012096	1.010356
16	1	0	4.774815	1.872686	-0.463323
17	1	0	3.146547	2.553818	-0.540148
18	6	0	-3.765089	1.797321	-0.057255
19	1	0	-3.146536	2.553820	-0.540165
20	1	0	-4.774808	1.872700	-0.463316
21	1	0	-3.810033	2.012117	1.010351

Triplet carbene from diethyl diazomalonate

Zero-point correction=	0.163782 (Hartree/Particle)
Thermal correction to Energy=	0.176492
Thermal correction to Enthalpy=	0.177436
Thermal correction to Gibbs Free Energy=	0.121672
Sum of electronic and zero-point Energies=	-573.630028
Sum of electronic and thermal Energies=	-573.617318
Sum of electronic and thermal Enthalpies=	-573.616374
Sum of electronic and thermal Free Energies=	-573.672138

Center Number	Atomic Number	Atomic Type	Coordinates (Angstroms) X	Y	Z
1	6	0	0.000000	-0.220824	-0.000008
2	6	0	-1.299054	0.336539	-0.290157
3	6	0	1.299052	0.336537	0.290152
4	8	0	-1.433542	1.394513	-0.874199
5	8	0	1.433538	1.394505	0.874207
6	8	0	2.297063	-0.455746	-0.110321
7	8	0	-2.297061	-0.455750	0.110311
8	6	0	3.649176	-0.002139	0.181692
9	1	0	3.749544	0.097126	1.262565
10	1	0	3.785970	0.981873	-0.265658
11	6	0	-3.649176	-0.002139	-0.181687
12	1	0	-3.749553	0.097136	-1.262558
13	1	0	-3.785965	0.981869	0.265672
14	6	0	-4.607418	-1.019637	0.385887
15	1	0	-4.492779	-1.110260	1.466140
16	1	0	-5.630749	-0.704350	0.177458
17	1	0	-4.455367	-2.000600	-0.064530
18	6	0	4.607421	-1.019634	-0.385882
19	1	0	4.492792	-1.110246	-1.466137
20	1	0	5.630751	-0.704350	-0.177441
21	1	0	4.455365	-2.000600	0.064525

Ethyl diazofluoroacetate (2d)

Zero-point correction=	0.096205 (Hartree/Particle)
Thermal correction to Energy=	0.105725
Thermal correction to Enthalpy=	0.106669
Thermal correction to Gibbs Free Energy=	0.060519
Sum of electronic and zero-point Energies=	-515.267622
Sum of electronic and thermal Energies=	-515.258101
Sum of electronic and thermal Enthalpies=	-515.257157
Sum of electronic and thermal Free Energies=	-515.303307

Center Number	Atomic Number	Atomic Type	Coordinates (Angstroms) X	Y	Z
1	6	0	-0.044456	-0.447145	0.000003
2	8	0	-0.142497	-1.657251	0.000003
3	8	0	1.094217	0.250482	0.000003
4	6	0	-1.199178	0.431376	-0.000002
5	6	0	2.326825	-0.523814	0.000006
6	1	0	2.331010	-1.161829	-0.883043
7	1	0	2.331008	-1.161825	0.883058
8	6	0	3.478531	0.450380	0.000005
9	1	0	3.459291	1.086104	0.885326
10	1	0	4.417475	-0.105131	0.000011
11	1	0	3.459297	1.086095	-0.885322
12	7	0	-2.398905	-0.066314	-0.000006
13	7	0	-3.443005	-0.497856	-0.000009
14	9	0	-1.120978	1.777238	-0.000004

Ethyl diazofluoroacetate TS

Zero-point correction=	0.092905 (Hartree/Particle)
Thermal correction to Energy=	0.102714
Thermal correction to Enthalpy=	0.103658
Thermal correction to Gibbs Free Energy=	0.056632
Sum of electronic and zero-point Energies=	-515.231693
Sum of electronic and thermal Energies=	-515.221885
Sum of electronic and thermal Enthalpies=	-515.220941
Sum of electronic and thermal Free Energies=	-515.267967

Center Number	Atomic Number	Atomic Type	Coordinates (Angstroms) X	Y	Z
1	6	0	0.113520	-0.689653	-0.247393
2	8	0	0.665678	-1.765317	-0.183566
3	8	0	0.703794	0.492076	-0.407840
4	6	0	-1.384801	-0.675784	-0.414803
5	6	0	2.158428	0.507278	-0.538052
6	1	0	2.355724	1.393445	-1.136859
7	1	0	2.463623	-0.377049	-1.092935
8	6	0	2.833689	0.586726	0.812979
9	1	0	2.631321	-0.299902	1.412490
10	1	0	3.913261	0.657971	0.669069
11	1	0	2.507025	1.468962	1.364094
12	7	0	-1.848698	1.074070	0.151934
13	7	0	-2.383345	2.016910	-0.007800
14	9	0	-1.947493	-1.407306	0.536674

Singlet carbene from ethyl diazofluoroacetate

Zero-point correction=	0.085123 (Hartree/Particle)
Thermal correction to Energy=	0.092993
Thermal correction to Enthalpy=	0.093937
Thermal correction to Gibbs Free Energy=	0.052250
Sum of electronic and zero-point Energies=	-405.686128
Sum of electronic and thermal Energies=	-405.678258
Sum of electronic and thermal Enthalpies=	-405.677314
Sum of electronic and thermal Free Energies=	-405.719001

Center Number	Atomic Number	Atomic Type	Coordinates (Angstroms) X	Y	Z
1	6	0	0.649122	0.367565	0.147826
2	8	0	0.796639	1.540071	-0.119599
3	8	0	-0.465667	-0.330222	0.179680
4	6	0	1.880481	-0.339355	0.632861
5	6	0	-1.716798	0.378113	-0.122540
6	1	0	-1.624461	0.809578	-1.117557
7	1	0	-1.823208	1.184889	0.601124
8	6	0	-2.839270	-0.623419	-0.035176
9	1	0	-2.909824	-1.052829	0.964116
10	1	0	-3.781992	-0.121310	-0.256869
11	1	0	-2.706114	-1.430504	-0.755517
12	9	0	2.484069	-0.862894	-0.405974

Triplet carbene from ethyl diazofluoroacetate

Zero-point correction=	0.085774 (Hartree/Particle)
Thermal correction to Energy=	0.093345
Thermal correction to Enthalpy=	0.094289
Thermal correction to Gibbs Free Energy=	0.052403
Sum of electronic and zero-point Energies=	-405.676476
Sum of electronic and thermal Energies=	-405.668905
Sum of electronic and thermal Enthalpies=	-405.667961
Sum of electronic and thermal Free Energies=	-405.709846

Center Number	Atomic Number	Atomic Type	Coordinates (Angstroms) X	Y	Z
1	6	0	0.597545	0.192344	-0.000001
2	8	0	0.778249	1.393200	0.000000
3	8	0	-0.581202	-0.434906	0.000000
4	6	0	1.689998	-0.765652	-0.000001
5	6	0	-1.766399	0.409850	0.000001
6	1	0	-1.733965	1.046885	-0.883584
7	1	0	-1.733964	1.046884	0.883586
8	6	0	-2.973484	-0.494581	0.000001
9	1	0	-2.991669	-1.129955	0.885621
10	1	0	-3.878042	0.115401	0.000002
11	1	0	-2.991670	-1.129954	-0.885620
12	9	0	2.940776	-0.407487	-0.000001

Ethyl chlorodiazoacetate (2a)

Zero-point correction=	0.094994 (Hartree/Particle)
Thermal correction to Energy=	0.103912
Thermal correction to Enthalpy=	0.104857
Thermal correction to Gibbs Free Energy=	0.060028
Sum of electronic and zero-point Energies=	-875.635442
Sum of electronic and thermal Energies=	-875.626523
Sum of electronic and thermal Enthalpies=	-875.625579
Sum of electronic and thermal Free Energies=	-875.670408

Center Number	Atomic Number	Atomic Type	Coordinates (Angstroms) X	Y	Z
1	6	0	0.145214	-0.669677	0.000001
2	8	0	0.148421	-1.877739	0.000004
3	8	0	1.236464	0.114283	0.000002
4	6	0	-1.075013	0.133558	-0.000001
5	6	0	2.523131	-0.564760	0.000002
6	1	0	2.575562	-1.200630	-0.883065
7	1	0	2.575559	-1.200637	0.883065
8	6	0	3.600419	0.491598	0.000008
9	1	0	3.535257	1.124040	0.885516
10	1	0	4.577583	0.006816	0.000007
11	1	0	3.535258	1.124048	-0.885494
12	7	0	-1.022856	1.433257	-0.000003
13	7	0	-0.983880	2.559330	-0.000006
14	17	0	-2.646686	-0.590495	-0.000005

Ethyl chlorodiazoacetate TS

Zero-point correction=	0.091485 (Hartree/Particle)
Thermal correction to Energy=	0.101664
Thermal correction to Enthalpy=	0.102608
Thermal correction to Gibbs Free Energy=	0.054336
Sum of electronic and zero-point Energies=	-875.590755
Sum of electronic and thermal Energies=	-875.580576
Sum of electronic and thermal Enthalpies=	-875.579632
Sum of electronic and thermal Free Energies=	-875.627904

Center Number	Atomic Number	Atomic Type	Coordinates (Angstroms) X	Y	Z
1	6	0	0.109498	-0.755614	-0.267799
2	8	0	0.187240	-1.951837	-0.458582
3	8	0	1.126221	0.051391	0.027136
4	6	0	-1.182311	-0.104675	-0.583039
5	6	0	2.450394	-0.559886	0.080187
6	1	0	2.627263	-1.074129	-0.863290
7	1	0	2.452524	-1.298344	0.881050
8	6	0	3.450441	0.542808	0.322769
9	1	0	3.256688	1.054253	1.265912
10	1	0	4.452454	0.114161	0.369288
11	1	0	3.431662	1.276963	-0.482932
12	7	0	-0.922778	1.718897	-0.036928
13	7	0	-1.008602	2.788647	-0.257187
14	17	0	-2.480984	-0.656349	0.413390

Singlet carbene from ethyl chlorodiazoacetate

Zero-point correction=	0.083998 (Hartree/Particle)
Thermal correction to Energy=	0.092054
Thermal correction to Enthalpy=	0.092998
Thermal correction to Gibbs Free Energy=	0.050099
Sum of electronic and zero-point Energies=	-766.047325
Sum of electronic and thermal Energies=	-766.039269
Sum of electronic and thermal Enthalpies=	-766.038324
Sum of electronic and thermal Free Energies=	-766.081224

Center Number	Atomic Number	Atomic Type	Coordinates (Angstroms) X	Y	Z
1	6	0	0.184927	0.587207	0.199369
2	8	0	0.191560	1.730072	-0.212012
3	8	0	-0.830845	-0.253915	0.234922
4	6	0	1.419040	0.130772	0.856072
5	6	0	-2.128293	0.236328	-0.241877
6	1	0	-2.026926	0.480143	-1.298334
7	1	0	-2.366142	1.146643	0.305944
8	6	0	-3.141248	-0.853510	-0.003750
9	1	0	-3.224049	-1.090941	1.056753
10	1	0	-4.117135	-0.514344	-0.354385
11	1	0	-2.881004	-1.761715	-0.547372
12	17	0	2.454293	-0.627872	-0.247340

Triplet carbene from ethyl chlorodiazoacetate

Zero-point correction=	0.084447 (Hartree/Particle)
Thermal correction to Energy=	0.092326
Thermal correction to Enthalpy=	0.093270
Thermal correction to Gibbs Free Energy=	0.050051
Sum of electronic and zero-point Energies=	-766.044853
Sum of electronic and thermal Energies=	-766.036974
Sum of electronic and thermal Enthalpies=	-766.036030
Sum of electronic and thermal Free Energies=	-766.079249

Center Number	Atomic Number	Atomic Type	Coordinates (Angstroms) X	Y	Z
1	6	0	0.106017	0.263991	0.000000
2	8	0	0.207319	1.475185	0.000012
3	8	0	-1.044338	-0.420646	-0.000002
4	6	0	1.240552	-0.634851	-0.000010
5	6	0	-2.267000	0.367223	0.000010
6	1	0	-2.264668	1.006113	-0.882979
7	1	0	-2.264660	1.006097	0.883011
8	6	0	-3.431339	-0.591997	0.000006
9	1	0	-3.420152	-1.227891	0.885380
10	1	0	-4.363255	-0.024741	0.000020
11	1	0	-3.420165	-1.227869	-0.885384
12	17	0	2.855275	-0.258484	-0.000010

Ethyl bromodiazoacetate (2b)

Zero-point correction=	0.094643 (Hartree/Particle)
Thermal correction to Energy=	0.104651
Thermal correction to Enthalpy=	0.105595
Thermal correction to Gibbs Free Energy=	0.056173
Sum of electronic and zero-point Energies=	-2989.633155
Sum of electronic and thermal Energies=	-2989.623147
Sum of electronic and thermal Enthalpies=	-2989.622203
Sum of electronic and thermal Free Energies=	-2989.671625

Center Number	Atomic Number	Atomic Type	Coordinates (Angstroms) X	Y	Z
1	6	0	0.625321	0.976884	0.000007
2	8	0	0.904076	2.158615	0.000013
3	8	0	1.503291	-0.026815	0.000004
4	6	0	-0.756178	0.500631	-0.000001
5	6	0	2.910216	0.343880	0.000014
6	1	0	3.105548	0.951091	0.883335
7	1	0	3.105555	0.951112	-0.883291
8	6	0	3.716605	-0.931093	0.000002
9	1	0	3.506474	-1.530925	-0.885684
10	1	0	4.779014	-0.683323	0.000008
11	1	0	3.506467	-1.530946	0.885671
12	7	0	-1.692744	1.404981	-0.000001
13	7	0	-2.507385	2.180760	-0.000001
14	35	0	-1.338196	-1.304383	-0.000008

Ethyl bromodiazoacetate TS

Zero-point correction=	0.090741 (Hartree/Particle)
Thermal correction to Energy=	0.101238
Thermal correction to Enthalpy=	0.102183
Thermal correction to Gibbs Free Energy=	0.052183
Sum of electronic and zero-point Energies=	-2989.585955
Sum of electronic and thermal Energies=	-2989.575458
Sum of electronic and thermal Enthalpies=	-2989.574514
Sum of electronic and thermal Free Energies=	-2989.624514

Center Number	Atomic Number	Atomic Type	Coordinates (Angstroms) X	Y	Z
1	6	0	-0.548874	0.820818	-0.055406
2	8	0	-0.609430	1.894732	-0.607604
3	8	0	-1.589689	0.017120	0.194854
4	6	0	0.637450	0.246582	0.650300
5	6	0	-2.911936	0.530483	-0.145685
6	1	0	-2.932358	0.730447	-1.216092
7	1	0	-3.057369	1.471881	0.382980
8	6	0	-3.925744	-0.510328	0.257870
9	1	0	-3.888189	-0.705321	1.329656
10	1	0	-4.925500	-0.149108	0.012084
11	1	0	-3.763033	-1.448141	-0.273316
12	7	0	1.982542	1.571030	0.061922
13	7	0	2.798738	2.258958	0.301876
14	35	0	1.233859	-1.386566	-0.106353

Singlet carbene from ethyl bromodiazoacetate

Zero-point correction=	0.083536 (Hartree/Particle)
Thermal correction to Energy=	0.091728
Thermal correction to Enthalpy=	0.092672
Thermal correction to Gibbs Free Energy=	0.048645
Sum of electronic and zero-point Energies=	-2880.043608
Sum of electronic and thermal Energies=	-2880.035416
Sum of electronic and thermal Enthalpies=	-2880.034472
Sum of electronic and thermal Free Energies=	-2880.078498

Center Number	Atomic Number	Atomic Type	Coordinates (Angstroms) X	Y	Z
1	6	0	0.490229	0.745486	0.214700
2	8	0	0.574076	1.839008	-0.311259
3	8	0	1.425501	-0.181469	0.301778
4	6	0	-0.749718	0.490605	0.953310
5	6	0	2.743037	0.137255	-0.258065
6	1	0	3.107770	1.036256	0.236665
7	1	0	2.617158	0.348173	-1.318737
8	6	0	3.638343	-1.051085	-0.016380
9	1	0	3.252345	-1.943280	-0.509409
10	1	0	4.628586	-0.839551	-0.422170
11	1	0	3.743731	-1.257059	1.048616
12	35	0	-2.002216	-0.358240	-0.123443

Triplet carbene from ethyl bromodiazoacetate

Zero-point correction=	0.083832 (Hartree/Particle)
Thermal correction to Energy=	0.091930
Thermal correction to Enthalpy=	0.092875
Thermal correction to Gibbs Free Energy=	0.048175
Sum of electronic and zero-point Energies=	-2880.041403
Sum of electronic and thermal Energies=	-2880.033305
Sum of electronic and thermal Enthalpies=	-2880.032360
Sum of electronic and thermal Free Energies=	-2880.077060

Center Number	Atomic Number	Atomic Type	Coordinates (Angstroms) X	Y	Z
1	6	0	-0.612571	0.306041	0.000003
2	8	0	-0.545892	1.519801	0.000011
3	8	0	-1.745309	-0.408746	0.000006
4	6	0	0.543088	-0.565237	-0.000008
5	6	0	-2.989526	0.344905	0.000018
6	1	0	-3.005075	0.983589	-0.883005
7	1	0	-3.005061	0.983584	0.883045
8	6	0	-4.125770	-0.647566	0.000024
9	1	0	-4.095698	-1.282914	0.885318
10	1	0	-5.073999	-0.108227	0.000037
11	1	0	-4.095716	-1.282906	-0.885276
12	35	0	2.306109	-0.137441	-0.000014

N2

Zero-point correction=	0.005582 (Hartree/Particle)
Thermal correction to Energy=	0.007943
Thermal correction to Enthalpy=	0.008887
Thermal correction to Gibbs Free Energy=	-0.012842
Sum of electronic and zero-point Energies=	-109.559414
Sum of electronic and thermal Energies=	-109.557054
Sum of electronic and thermal Enthalpies=	-109.556110
Sum of electronic and thermal Free Energies=	-109.577839

Center Number	Atomic Number	Atomic Type	Coordinates (Angstroms) X	Y	Z
1	7	0	0.000000	0.000000	0.545399
2	7	0	0.000000	0.000000	-0.545399
